# Exploring the identification of multiple bacteria on stainless steel using multi-scale spectral imaging from microscopic to macroscopic

**DOI:** 10.1038/s41598-022-19617-3

**Published:** 2022-09-14

**Authors:** Jun-Li Xu, Ana Herrero-Langreo, Sakshi Lamba, Mariateresa Ferone, Anastasia Swanson, Vicky Caponigro, Amalia G. M. Scannell, Aoife A. Gowen

**Affiliations:** 1 School of Biosystems and Food Engineering, University College Dublin, Belfield, Dublin, Ireland; 2 School of Agriculture and Food Science, University College Dublin, Belfield, Dublin, Ireland; 3 Institute of Food and Health, University College Dublin, Belfield, Dublin 4, Ireland; 4 Conway Institute, University College Dublin, Belfield, Dublin 4, Ireland; 5 Department of Pharmacy, University of Salerno, Via Giovanni Paolo II, 132, 84084 Fisciano, Italy

**Keywords:** Microbiology, Analytical chemistry

## Abstract

This work investigates non-contact reflectance spectral imaging techniques, i.e. microscopic Fourier transform infrared (FTIR) imaging, macroscopic visible-near infrared (VNIR), and shortwave infrared (SWIR) spectral imaging, for the identification of bacteria on stainless steel. Spectral images of two Gram-positive (GP) bacteria (*Bacillus subtilis* (BS) and *Lactobacillus plantarum* (LP)), and three Gram-negative (GN) bacteria (*Escherichia coli* (EC), *Cronobacter sakazakii* (CS), and *Pseudomonas fluorescens* (PF))*,* were collected from dried suspensions of bacterial cells dropped onto stainless steel surfaces. Through the use of multiple independent biological replicates for model validation and testing, FTIR reflectance spectral imaging was found to provide excellent GP/GN classification accuracy (> 96%), while the fused VNIR-SWIR data yielded classification accuracy exceeding 80% when applied to the independent test sets. However, classification within gram type was far less reliable, with lower accuracies for classification within the GP (< 75%) and GN (≤ 51%) species when calibration models were applied to the independent test sets, underlining the importance of independent model validation when dealing with samples of high biological variability.

## Introduction

Bacterial contamination in food products is a public concern globally, due to its association with increased food waste and foodborne illness. Food contact surfaces, which represent all surfaces in contact with food products during production, processing, packaging, and storage, are potential sources of contamination^1^. Attachment of bacteria to equipment surfaces can transmit pathogens to food, particularly when the microorganisms concerned are capable of forming biofilms^2,3^. In this context, the development of effective detection methods for microbial contamination is of significant importance to the food industry, to reduce food contamination, thereby protecting consumers from exposure to bacteria that cause foodborne illnesses. Conventional standard methods for the detection of bacterial species are based on microbiological culturing and isolation of the pathogen which is subsequently identified by biochemical and/or serological tests. However, these methods are laborious, requiring sample destruction or swabbing, time-consuming and unsuitable for rapid detection and discrimination of bacteria attached on the surfaces of equipment. Therefore a need exists to develop fast, reliable, low-cost, and non-destructive analytical methods to detect and/or identify bacterial contamination on the surfaces^4^.

Spectral imaging, a broad term encompassing optical techniques that combine spectroscopy and imaging, has recently emerged as an advanced measurement technique in the food industry, primarily for monitoring quality and safety attributes of food products, and its potential for microbial characterisation has been suggested^5^. Mid-infrared spectra, commonly obtained using the Fourier Transform Infrared (FTIR) technique, contain information on the fundamental vibrational modes of functional groups within molecules of biological samples. FTIR micro-spectroscopic imaging in transmission mode has previously been applied to the task of microbial identification with promising results for bacterial suspensions^6–8^ or microcolonies^9^ transferred and dried onto IR transparent substrates. Unfortunately, many reports from the literature lack model validation with independent biological replicates, which is essential considering the biological variability routinely encountered in microbiological experiments. A notable (though not exclusive) exception to this is the recent study by Lasch et al.^9^, where FTIR transmission imaging was used for identification of multiple GP and GN strains and at least 3 biological replicates were tested for each strain. In this landmark study, the authors developed a method comprising of cultivation of microcolonies of microorganisms for 6–24 h, followed by transferring the microcolonies’ upper cell layers onto IR transparent CaF_2_ windows using a custom-designed stamping device. This was followed by FTIR transmission imaging and classification with a neural network. Compared to the conventional agar plate culturing method, the proposed method in^9^ consists of a relatively short cultivation step. However, CaF_2_ windows are usually fragile and costly, and therefore, they are not easily applicable in an industrial setting. In another recent study, Martak et al. demonstrated the utility of FTIR spectroscopy for the typing of clinical isolates of Gram-negative bacilli clones using the lower cost Silicon substrates for transmission measurement^10^. However, the IR transmission modality approach does not satisfy the considerably more challenging goal of bacterial identification on surfaces of relevance to the food industry. Towards this goal, we have recently probed the capability of FTIR reflectance micro-spectroscopic imaging to detect and classify bacterial cells that were dried onto metallic surfaces, i.e. Aluminium, Stainless Steel 304 and 316^11^. In our previous work, we found that GP *Bacillus subtilis* and GN *Escherichia coli* could be reliably detected and distinguished from each other on both aluminium and stainless-steel surfaces at pre-application optical densities ranging from 10 to 0.1 OD_600_.

Spectral information in the near-infrared (NIR) or short-wave infrared (SWIR) wavelength ranges, originates from overtones and combinations of the fundamental vibrations of molecular bonds within a sample, typically found in the MIR range, and therefore can be used to acquire knowledge on chemical composition. Compared to conventional FTIR microscopic systems, macroscopic visible-NIR (VNIR, approximately 400–1000 nm) or SWIR (approximately 1000–2500 nm) spectral imaging systems are advantageous in terms of data acquisition speed. For example, in the present study, microscopic FTIR imaging required approximately 20 min per drop with a coarse resolution of 200 μm per pixel, while macroscopic VNIR or SWIR imaging could obtain a full spectral image the same spatial region within 1 min. This enhanced speed enables the rapid scanning of large areas and are therefore considered more practical for real-life applications, such as in the detection of disease on crops^12,13^.

Macroscopic spectral imaging coupled with chemometrics has been applied in numerous studies to classify bacterial colonies grown on agar, as summarised in a recent review^5^. Of particular note is a study on the use of VNIR macroscopic imaging for the classification of colonies of pathogenic bacteria related to urinary tract infections, cultured on blood agar^14^, in which a variety of chemometric approaches were evaluated to classify colonies at the colony level. More recently, spectral imaging has been developed for the identification and quantification of lentiviral particles in fluid samples^15^ and for primary screening at the point-of-care of SARS-CoV-2^16^.

When analysing spectral imaging data, it is generally advantageous to utilise the information from multiple wavelengths simultaneously using multivariate chemometric approaches. Partial least squares-discriminant analysis (PLS-DA) is widely used in classification problems due to its interpretability and capability of handling multicollinearity problems in high dimensional data^17^. PLS-DA models separate classes according to linear boundaries, which is sufficient for linearly separable data; for non-linear data support vector machine (SVM) models have the advantage of more complex curved boundaries between classes, however, the risk of model overfitting must be avoided through the use of appropriate cross and external data validation^18^. In recent years, deep learning (DL) models have been introduced for spectral imaging, predominantly in the remote sensing domain^19^, but more recently in vibrational spectral imaging studies for bacterial detection^20^. DL models can extract hidden and sophisticated structures/features (both linear and non-linear features) contained in the raw data. They also support the extraction of both spectral and spatial features of spectral imaging data and allow flexibility in network architectures making DL models attractive and powerful for spectral image data analysis^21^; however, they lack the interpretability offered by less computationally demanding approaches such as PLS-DA.

This work builds on our previous findings by investigating the performance of microscopic FTIR reflectance imaging as compared to macroscopic VNIR and SWIR spectral imaging for classification of multiple bacterial species, i.e., *Bacillus subtilis*, *Lactobacillus plantarum*, *Escherichia coli*, *Cronobacter sakazakii,* and *Pseudomonas fluorescens* on stainless steel 316 surfaces. The performance of object and pixel-wise classification is evaluated at two levels: 1) differentiation of GP and GN; and 2) classification of individual bacterial species within the same Gram type.

## Material and methods

### Sample preparation

The bacteria species used in this study, representing a selection of GP and GN bacteria, were: *Bacillus subtilis (B. subtilis)* DSM 10, *Lactobacillus plantarum (L. plantarum)* DSM 20174, *Escherichia coli (E. coli)* DSM 11250, *Cronobacter sakazakii (C. sakazakii)* ATCC 29544*,* and *Pseudomonas fluorescens (P. fluorescens)* DSM 50090, respectively labelled as BS, LP, EC, CS and PF for the sake of simplicity. These particular bacterial species were chosen to represent relevant bacteria in the dairy industry. The bacteria were provided by the German Collections of Microorganisms and Cell Cultures (Braunschweig, Germany), except *C. sakazakii* which was purchased from the American Type Culture Collection (ATCC, Manassas, Virginia, United States).

The bacterial suspensions were prepared from − 80 °C glycerol stock by suspending in 4 mL of Tryptic Soya Broth (TSB;Oxoid, CM0129). Bacteria precultures were grown overnight at 30 °C followed by growth in fresh TSB to reach the mid-exponential phase. Cells were harvested by centrifugation (5000 rpm for 15 min at 4 °C) and washed twice in sterile phosphate buffer saline (Gibco, Life Tech. 18912-014), followed by two washing steps in sterile water. The concentration of cell suspensions was assessed by optical density (OD) measurements (Shimadzu UVmini Spectrophotometer Model 1240) at a wavelength of 600 nm (OD_600_).

For image collection, duplicate 10 µL bacterial suspensions corresponding to 10 OD were deposited on stainless steel (STS) slides (AISI 316 finished 2B, purchased from Amari Ireland Ltd., Dublin, Ireland), dried for 20–30 min in a safety cabinet at room temperature, and stored at 4 °C before scanning. Prior to this, STS slides were washed with a solution of ethanol and acetone (1:1 v/v) to remove any trace of adhesive. Each coupon was immersed for 10 min in the acetone/ethanol solution and was then rinsed for 5 min with deionised (DI) water, followed by sterilization in autoclave at 121 °C for 20 min. For evaluation of reproducibility and replicability, 16 biological replicates of each species were prepared on different dates spanning 5 months, as reported in Table 1 (replicate was abbreviated to rep). Model calibration, validation and testing were carried out with samples prepared during this time range. As a further test of model robustness, 4 additional biological replicates were prepared and measured in June 2022 and models developed on the calibration set were applied to these new data points. Two drops of each replicate were placed on the same STS slide. Independent slides were prepared for FTIR and macroscopic imaging, in order to avoid any potential sample modifications due to illumination during VNIR or FTIR measurement. For the biological replicates obtained in June 2022 (i.e. reps 17–20 in Table 1), six drops were applied to each slide for macroscopic imaging.

**Table 1:**
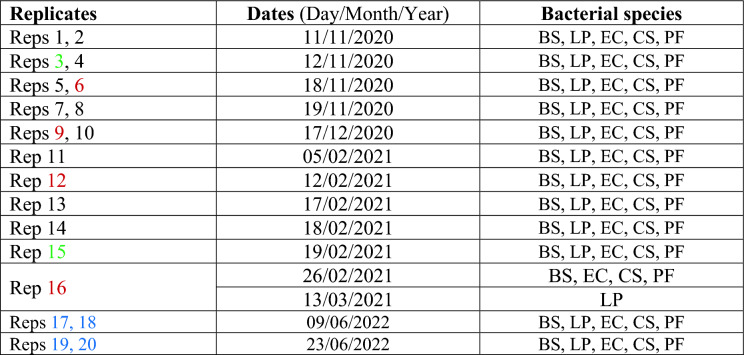
Details regarding sample replicates deposited on stainless steel.

Rep: Replicate*.* Replicates marked in green colour are samples used in validation set and red colour are used in the first test set (‘Test1') and blue colour are used in the second test set (‘Test2'). FTIR images of replicate 2 were not successful because of malfunction of the imaging system and for this reason are omitted from further analysis. BS: *Bacillus subtilis*; LP: *Lactobacillus plantarum*; EC: *Escherichia coli*, CS: *Cronobacter sakazakii*; PF: *Pseudomonas fluorescens.*

The plate count method was also applied to estimate the number of viable cells in the bacterial suspensions. In detail, resuspended washed cells were serially diluted to 10^–8^ in sterile water, 100 µL of which was then cultivated in duplicate on Tryptic Soy Agar. After incubation at 37 °C for 24 h, the number of colony-forming units (CFU) per plate was counted and converted to CFU/mL. As is conventional practice, colony counts in the range of 30–300 CFU were considered.

### FTIR image acquisition

FTIR imaging measurements were carried out using a Thermo Scientific Nicolet iN10 Infrared Microscope. This IR microscope is equipped with a liquid nitrogen cooled mercury-cadmium-tellurium (MCT) detector and a 10 × objective. The instrument was purged by gas nitrogen overnight before scanning. Spectral images were recorded in reflectance mode with 4 cm ^−1^ spectral resolution in the 4000–675 cm^−1^ range, taking the average of 4 scans per pixel. The aperture size and step size were 200 × 200 µm and 200 µm. The full bacterial drop was imaged with the actual drop size ranging from 3.4 mm × 3.4 mm to 6.4 mm × 6.0 mm. The background spectrum was collected every 10 min from a gold disc integrated into the standard sample plate.

### Macroscopic spectral image acquisition

Two spectral images were captured simultaneously for each bacterial sample using the same instrument installed with two spectral imaging cameras (HySpex by NEO Ltd., Oslo, Norway): one recording spectral data in the visible-near-infrared (VNIR) (HySpex VNIR-1800 camera) (406–997 nm) range; the other capturing the short-wave infrared (SWIR) (HySpex SWIR-384 camera) range (951–2496 nm). The nominal spatial resolution was 160 µm $\times$ 160 µm per pixel for VNIR and 730 µm $\times$ 730 µm per pixel for SWIR images. Two custom-made Hyspex lamps (12 V DC, 150 W), providing illumination in the spectral range 400–2500 nm were mounted symmetrically at ~ 45° to the vertical axis (perpendicular to the scanning direction). Samples were scanned with an integration time of 12,500 µs in the VNIR range and 8000 µs in the SWIR range. A Spectralon diffuse reflectance reference target (at reflectance of 50%) was included in every image to standardize spectra. Relative reflectance was obtained by normalizing radiance images by the radiance values of the Spectralon included in each image. Specifically, each pixel was normalized by the averaged Spectralon radiance value located on the same push-broom pixel position. True reflectance was then calculated by multiplying the relative reflectance values by the reference spectra of the Spectralon as provided by the supplier.

### Data analysis

#### Data pre-processing

All spectral data analysis and modelling were performed in the MATLAB computing environment (release R2020a, The MathWorks, Inc., Natick, MA, USA) incorporating functions from Statistics and Machine Learning Toolbox, Image Processing Toolbox and additional functions written in-house.

##### Background removal, extraction of spectra and exploratory PCA

Image segmentation is an essential and fundamental step in spectral image analysis, as the accuracy of the subsequent modelling is highly influenced by this process. In this work, the segmentation task for background removal consists of separating the bacterial pixels from the stainless steel pixels. Principal component analysis (PCA), one of the most widely used unsupervised techniques for spectral imaging analysis, enables reduction of the many spectral dimensions to a smaller number of principal components (PC) scores which capture the maximum variation in the data^22^. PCA was applied to each spectral image, and it was found that the PC1 score image highlighted the major differences between the stainless steel and bacterial pixels. Thus, manual thresholding of PC1 was performed to create a mask for the segmentation of the bacterial region. Only pixels within the mask representing bacteria were extracted and utilized for the following modelling process. In addition, to reduce data load, VNIR and SWIR images were cropped to include only the region of two bacterial drops, excluding the ink labels on the STS slides (see Fig. S1). Subsequently, mean or pixel spectra from the drops were extracted for exploratory analysis and classification model building.

For initial data exploration, PCA was applied to the combined mean spectra of each sample in the calibration set, for each modality. Spectra of the validation and test set were projected along the PC loading to produce the score values.

Spectral pre-treatment is important in chemometric data analysis because it can remove unwanted variation, such as instrumental and experimental artifacts, and thereby the pre-processed spectra are often better suited to the data analysis goals^23^. In addition, spectral pretreatments can be used to enhance visualisation of spectra; for this purpose, for the FTIR spectra, penalised asymmetric least squares smoothing^24,25^ was used to correct the baseline of the mean FTIR spectra, therefore providing an improved visual comparison of spectra among bacterial species. In essence, a baseline was first estimated in an iterative manner and subsequently subtracted from the original spectra. The smoothing and weighting (penalizing) parameters respectively were set as 10^6^ and 0.005. In addition, second derivative spectra (window size = 15 points and the polynomial order = 3) were applied to the mean spectra to identify overlapped absorption bands, for better visualization of absorption peaks.

##### VNIR and SWIR image registration

Since the spatial resolutions of VNIR and SWIR images are not the same, it is necessary to perform image registration to allow for data fusion at the pixel level. To achieve this, a geometric transformation was applied by selecting four pairs of matching control points to align the moving images with the fixed image (see example shown in Fig. S1). The SWIR image was chosen as the fixed image, while the VNIR image was considered as a moving image, which means that VNIR spectral images were registered to match the same spatial size of SWIR images. Four matching control points (labelled as 1, 2, 3, and 4) were selected on the fixed image, and four matching corresponding points (1’, 2’, 3’, and 4’) selected on the moving image. These four matching points were selected on the corners of the STS coupon and then labelled clockwise starting from the top left corner to the bottom left. Based on these four pairs of matching control points, an affine transformation was subsequently applied to align the two images by registering the moving image to the fixed one. An affine transformation maps variables, e.g. pixel intensity values located at position ($${x}_{1},{y}_{1})$$ in an input image, into new variables, i.e.,. ($${x}_{2},{y}_{2}$$) in an output image, by applying a linear combination of translation, rotation, scaling, and/or shearing (non-uniform scaling in some directions) operations. As can be seen in Fig. S1, after geometric transformation, the registered VNIR image has the same size as that of the SWIR image. More importantly, the four matching control points (labelled as 1, 2, 3, and 4) are located right on the corners of the stainless steel coupon in the registered VNIR image, further confirming the effectiveness of the geometric transformation. The same transformation was performed at each wavelength of the VNIR spectral image and subsequent data processing was performed on the registered VNIR image, in order to facilitate a fair comparison to the performance of the SWIR image.

#### Classification model development

To compare different models in an unbiased way, the 16 biological replicates were partitioned into training, validation, and test sets. Specifically, replicates 1, 2, 4, 5, 7, 8, 10, 11, 13, and 14 formed the training set, replicates 3 and 15 formed the validation set, and replicates 6, 9, 12, and 16 formed the first test set (‘Test1’) while replicates 17–20 formed the second independent test set (‘Test2’). Test set 1 consisted of 4 replicates of each of the 5 species, corresponding to 4 × 5 = 20 independent samples; 2 droplets from each replicate were used, providing a total of 40 droplet images. Considering the second independent test set (‘Test2’) the FTIR data consisted of 4 replicates of each species, corresponding to 4 × 5 = 20 independent samples; 2 droplets from each replicate were used, providing a total of 40 droplet images. In contrast for the macroscopic VNIR and SWIR data in Test 2, 6 drops per replicate were used, resulting in a total of 120 droplet images. From the first 16 replicates, the data partition was based on the simple rule that every third replicate plus Rep 16 were taken out of the training set. For the FTIR data analysis, replicate 2 was excluded from the calibration set due to equipment malfunctioning on that day (as mentioned in the footnote of Table 1). Models were assessed at two levels: (1) differentiation between GP and GN bacterial species, and (3) discrimination between individual bacterial species.

Prior to classification model development, pixel spectra were extracted from the spectral images after eliminating background and concatenated to form a matrix (X), while a label matrix (Y) was generated by labelling the corresponding pixel into a certain class. Data fusion of the VNIR and SWIR data was performed by horizontally concatenating the spatially registered VNIR and SWIR spectra at the pixel level. The performance of each classifier was evaluated by the overall classification accuracy, i.e., the percentage of samples correctly classified, and the mean accuracy per class was also computed to account for models in which there was not an identical number of spectra in each class. In addition to these metrics, classification maps were generated by applying the developed models to the spectral imaging data and were displayed to visualize the distribution of correctly and incorrectly classified pixels.

Discriminant models were constructed by using partial least squares-discriminant analysis (PLS-DA) and support vector machine (SVM) classifiers. PLS-DA and SVM models were applied both for object-level classification (using the mean drop spectra) or pixel-level classification (using the pixel spectra obtained after background removal). Models were constructed using untreated spectra and six spectral pre-treatments: SNV, 1st derivative Savitzky Golay pre-treatment (‘1st der’, window size = 15 points, polynomial order = 3), 2nd derivative Savitzky Golay pretreatment (‘2nd der’, window size = 15 points, polynomial order = 3), combinations of SNV followed by 1st or 2nd derivative pre-treatment and multiplicative scatter correction (MSC).

For PLS-DA modelling, parameters such as optimal pre-treatment and number of latent variables were selected by random cross validation on the data from the calibration set in which 70% of the spectra were randomly selected for model building and the remaining 30% were used for cross validation (the ‘randperm’ function in MATLAB was used to randomly permute the data). This process was repeated 100 times and the global and mean class accuracies, sensitivity, and specificity were calculated for each combination of spectral pre-treatment and number of latent variables. The optimal spectral pre-treatment and number of latent variables were selected based on consideration of the mean class accuracy, and product of sensitivity and specificity averaged over the 100 random folds on the calibration set. The optimal models were then applied to the validation set, from which the best spectral range/modality was determined. Finally, the optimal models were evaluated in terms of global and mean class accuracy on the independent test set.

While SVM and PLS-DA modelling inherently carry out binary classification, there are various procedures for extending them to multiclass problems. For discrimination of the 3 GN bacterial species, nPLS-DA and multiclass SVM with error correcting output codes (ECOC) were implemented.

In addition to this, for classification tasks that resulted in a low accuracy with PLS-DA and SVM models, a hybrid approach of PCA and LSTM (PCA-LSTM) proposed by^21^ was also employed, as follows: PCA was first performed on the global training dataset to produce loadings and scores. The first 5 PCs, explaining more than 95% variance, were selected and the score values were utilized as the input for LSTM. For validation and test sets, pixel spectra were projected along with the first 5 PC loadings by matrix multiplication producing score values to feed into the LSTM network. The structure of the LSTM network consists of a sequence input layer, two blocks containing a bidirectional long short-term memory (BiLSTM) layer and a dropout layer, a fully connected (FC) layer, a softmax layer, and finally an output layer. Details of the entire set of training options are provided in Table S1 and Fig. S2. To avoid overfitting during LSTM training, we applied early termination via setting the validation patience to 10, meaning that the training stops when it reaches 10 times that the loss on the validation set is not lower than the previously smallest loss.

## Results and discussion

### Spectral profiles and exploratory analysis

#### FTIR microscopic spectra

**Figure 1:**
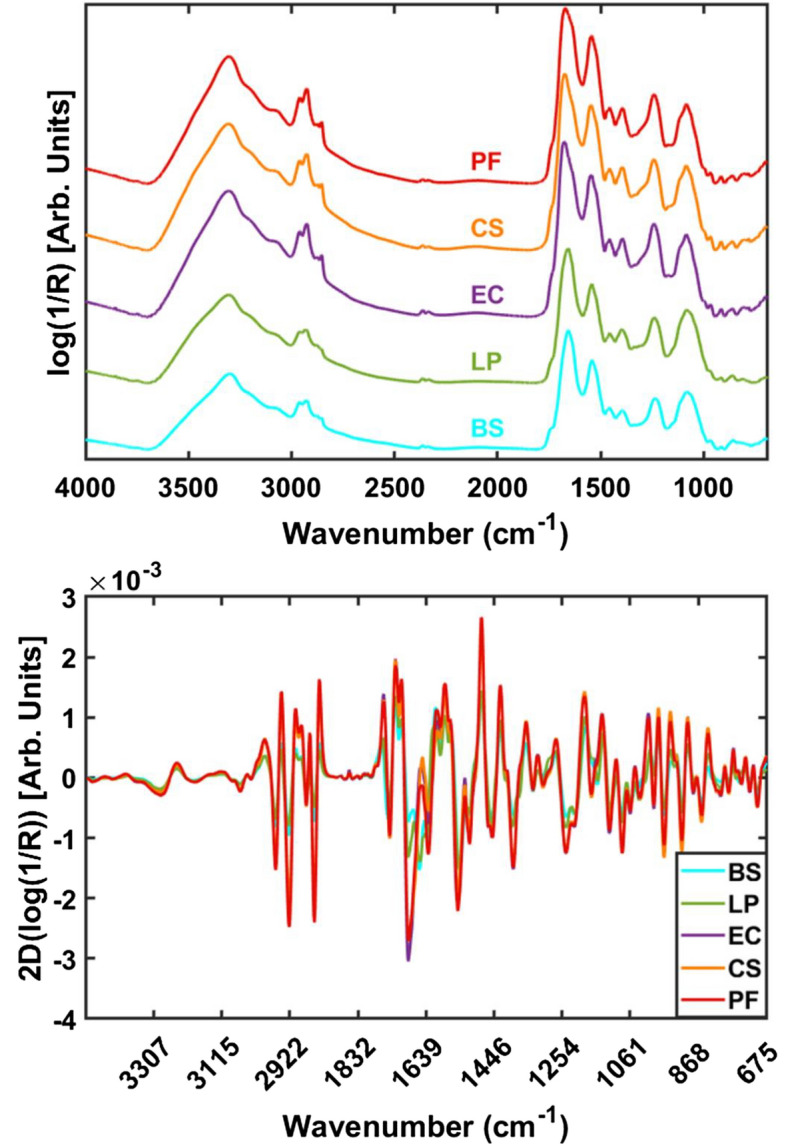
(Top panel): Mean FTIR spectra (after applying asymmetric least squares smoothing to remove baseline) of each bacterial species. The offset is manually added for better visualisation. (Bottom panel): Resultant FTIR spectra after applying the second derivative (window size = 15 points and the polynomial order = 3) on the mean spectrum of each bacterial species. R = Reflectance. Arb.units = Arbitrary units. BS: *Bacillus subtilis*; LP: *Lactobacillus plantarum*; EC: *Escherichia coli*, CS: *Cronobacter sakazakii*; PF: *Pseudomonas fluorescens.*

Figure 1 shows the mean spectra of all bacterial species in the training set (the number of pixels can be found in Table S4) investigated after applying asymmetric least squares smoothing to correct the baseline. Since the acquired mean FTIR spectra are a superposition of contributions from diverse biomolecules present in a cell, the spectral bands are observed to be broad and difficult to distinguish, which makes assessment of the specific contribution from any particular biomolecule rather challenging. As can be seen, the mean spectral profiles of bacterial species have a resemblance to each other, suggesting the similarity of the functional group chemistry of the selected bacterial species. A broad band covering 3700 cm^−1^ to 3000 cm^−1^ is mostly due to contributions from O–H and N–H stretching. A series of peaks can be noticed in the 3000–2800 cm^−1^ range due to C–H stretching coming from fatty acids^8,26^. This spectral region corresponds to the ‘‘lipid region’’ as it reflects information mostly from membrane lipids and some side chains of amino acids, since this region is dominated by C–H symmetrical or asymmetrical stretching vibrations of –CH_3_ and –CH_2_ functional groups^7^.

The next prevalent features in these spectra correspond to the ‘‘protein region’’ which relates to Amide I and Amide II vibrations of proteins in the range of 1700–1500 cm^−1^. As can be seen in Fig. 1, Amide I band positions are distinctive between GP and GN species. Specifically, the position of the Amide I band shifts from a position around 1672 cm^−1^ for GN species (i.e., 1674 cm^−1^ of *E. coli*, 1670 cm^−1^ of *C. sakazakii,* and 1672 cm^−1^ of *P. fluorescens*) to 1657 cm^−1^ for GP species i.e., *B. subtilis and L. plantarum*. To further enable the separation of overlapping bands, second derivative spectra (window size = 15 points and the polynomial order = 3) were obtained, as presented in Fig. 1 (lower panel). Notably, all bacterial species exhibit a major band at 1689 cm^−1^, while an absorption band at 1659 cm^−1^ is exclusively observable for GP species. An intense band centered at 1541 cm^−1^ representing N–H bending and C–N stretching in amide (Amide II)^27^ is obvious from all bacterial species. Another pronounced band that appears at 1240 cm^−1^ (Fig. 1) can be ascribed to asymmetric stretching vibrations of P = O related to phospholipids^8^.

Spectral features in the range of 1200–900 cm^−1^ are dominated by a combination of polysaccharides in the cell wall and phosphate-containing compounds, possibly nucleic acids from bacteria whose membrane has been damaged due to stretching vibrations of C–O–C, C–O–P, and PO_2_^−^ groups^28^. Within this range, a notable difference between GP and GN bacterial species can be perceived from the second derivative spectra of Fig. 1. In more detail, GN bacteria demonstrate a strong absorption at 1171 cm^−1^ and trivial absorption at 1153 cm^−1^, whereas GP bacteria show an intense absorption at 1153 cm^−1^ and minor feature at 1171 cm^−1^. This spectral distinction is linked to phosphate groups in bacteria according to^29^. GP bacteria have a thick peptidoglycan layer lacking an outer lipid membrane, with two other important constituents of cell walls being teichoic and teichuronic acids,. bacterial copolymers of glycerol phosphate. The cell envelope of GN bacteria is more complex. It is comprised of lipid outer membrane and a thin peptidoglycan which does not contain either teichoic or teichuronic acids.

Given the physical and biochemical dissimilarity between the GP and GN bacterial groups, it is reasonable to infer that the differing contributions of phosphate groups could serve as a point of spectral differentiation between the two bacterial groups.

It was also observed that the overall absorption is stronger for GN bacteria than that of GP bacteria (Fig. 1), possibly due to the higher cell counts in GN bacterial species (see Table S2). Taken together, although FTIR spectral profiles suggest the similarity of the functional group chemistry of bacteria, there is a clear spectral difference between GP and GN bacteria, which can be used in distinguishing them.

To examine reproducibility, the mean spectrum of each sample replicate after baseline correction was obtained and is plotted in Fig. S3. The general spectral features are similar over the whole spectral range, yet a considerable spectral variation among replicates is noticeable, indicating compositional and/or structural changes. Particularly, bacterial samples (reps 1–6) prepared on dates of November 11, 12, 18 2020 exhibited a much weaker absorption compared to the remaining samples. In addition to the difference in the intensity of absorption, some peak positions are also evidenced to be dissimilar among replicates of the same type of bacterial species, indicating the compositional changes in bacterial cells cultured from different experiments, which ultimately increases the difficulty for the classification modelling process. This is probably due to the highly complex, dynamically changing microbial environment, cellular activities, variations in the age of the bacterial population, and cell to cell relationships, contributing to the variations in the types and levels of proteins and metabolites present among experimental replicates. Table S2 demonstrates that samples of reps 1–4 had generally a lower cell count for individual bacterial species, possibly due to a difference between samples in which the bacterial populations exhibit a different mean “age”, meaning that the samples with a lower count probably contain more dead bacteria. However, this does not necessary explain differences in peak intensity, as dead cells should also give a spectral signal.

#### VNIR and SWIR macroscopic spectra

**Figure 2:**
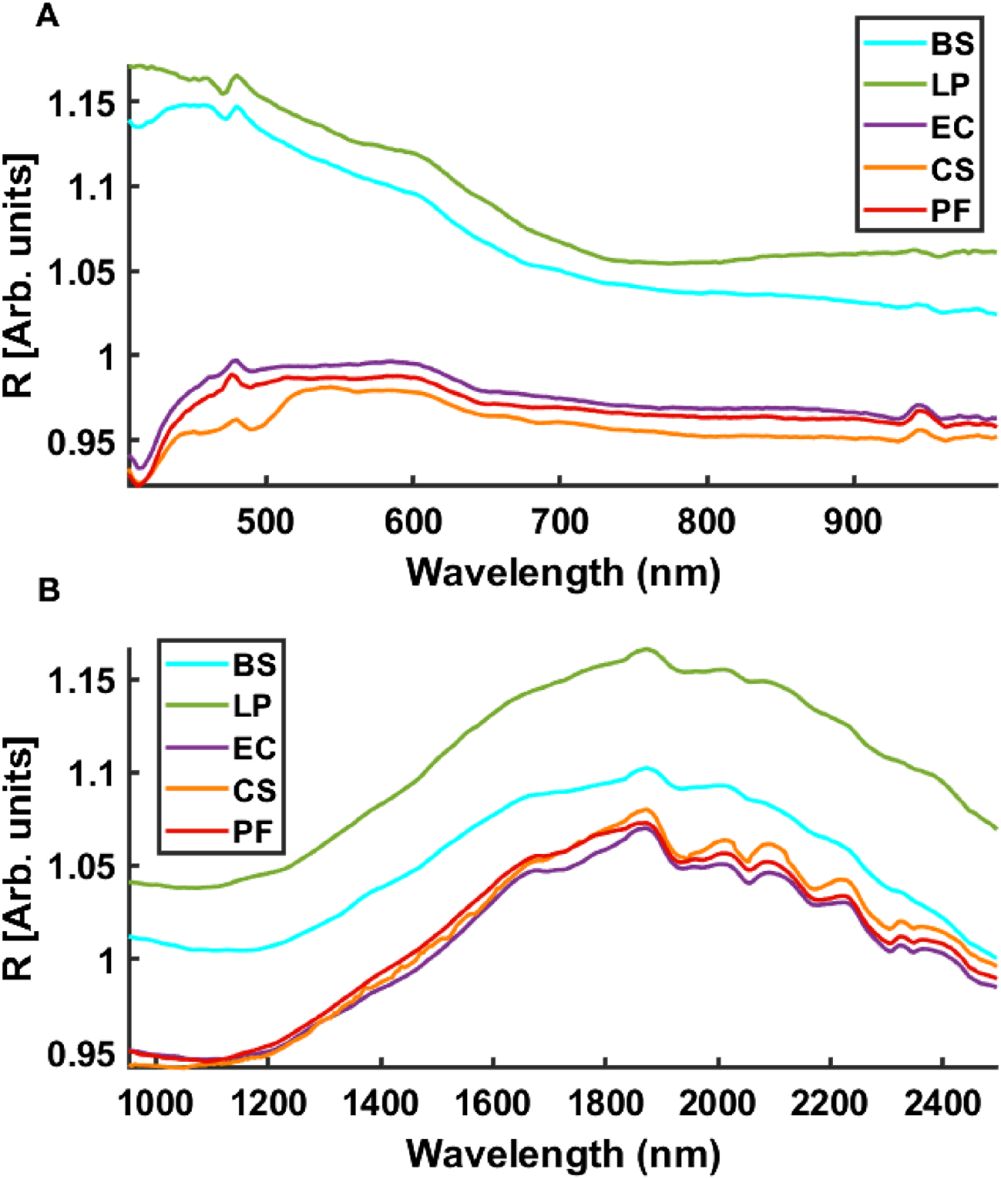
Mean VNIR (**A**) and SWIR (**B**) spectra of all bacterial species collected from stainless steel. R = Reflectance. Arb.units = Arbitrary units. BS: *Bacillus subtilis*; LP: *Lactobacillus plantarum*; EC: *Escherichia coli*, CS: *Cronobacter sakazakii*; PF: *Pseudomonas fluorescens.*

Mean spectra of individual bacterial species in the training set (the number of pixels can be found in Table S4) collected from the suspensions dried on stainless steel are shown in Fig. 2. As can be seen, the spectral shapes over the entire spectral range are distinctive between GP, i.e., *B. subtilis* and *L. plantarum*,and GN types, i.e., *E. coli*, *C. sakazakii* and *P. fluorescens*. In this study, the GP species exhibited higher reflectance, suggesting the potential to discriminate these two groups. It can also be noted that there are no major differences in the spectral features obtained from bacteria species with the same Gram type.

To evaluate the spectral variation among different biological replicates, the mean spectrum of each replicate, consisting of two bacterial suspension drops deposited on the same STS slide, was computed and is exhibited in Fig. S4. High variability in the spectra collected from all replicates is evidenced. In particular, replicates 1–6 are observed to present a different spectral trend compared to the remaining replicates, which is consistent with FTIR spectral profiles (see Fig. S3). Although care was taken to ensure that the same experimental protocol was used for each replicate, there are still variations in bacterial cells cultured from different experiments, related to uncontrolled variations in laboratory conditions during sample preparation (such as temperature and relative humidity) which will pose challenges for the subsequent classification. This high spectral variability contrasts with the high spectral reproducibility over three independent biological replicates reported by^9^, in which FTIR spectra were collected from cultivated bacterial colonies, transferred onto IR transparent slides; however, as the authors of this study point out, the need for strict standardisation of cultivation and sample preparation conditions is a drawback for the practical application of this technique. In contrast, in a study of suspensions of *Salmonella* colonies using darkfield VNIR microscopic spectral imaging^30^, no significant difference in *S.typhimurium* spectra was found using four different agar types and 4 different incubation temperatures; however incubation pH was found to influence the spectra shape measured in the wavelength range 400–800 nm.

#### Principal components analysis applied to mean spectra

**Figure 3:**
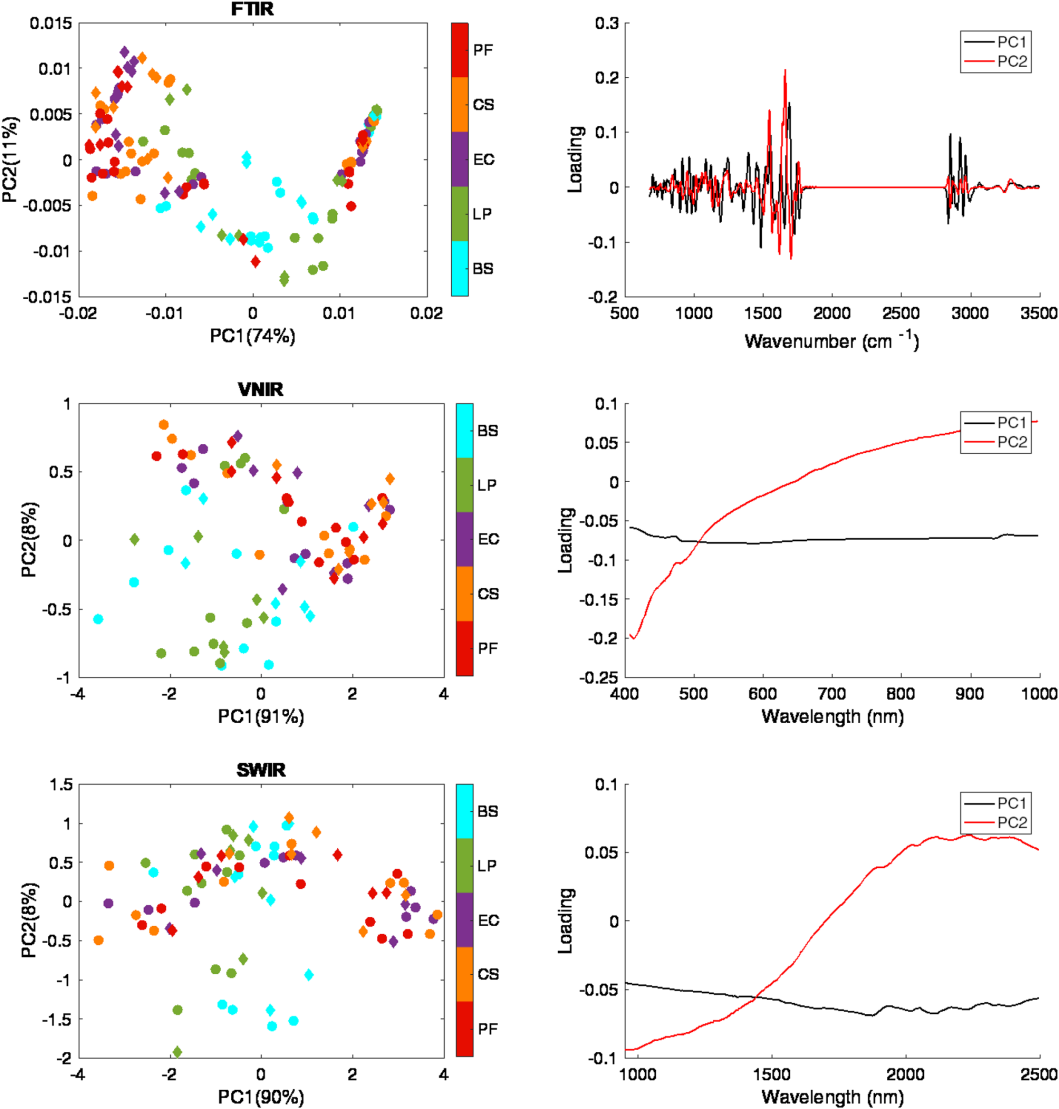
Score and loading plots (PC1 and PC2) of the PCA model built from the calibration sets of mean spectra for each modality. Note: A circle marker refers to the observation in the training set, while a diamond marker denotes the sample in the validation or test set. BS: *Bacillus subtilis*; LP: *Lactobacillus plantarum*; EC: *Escherichia coli*, CS: *Cronobacter sakazakii*; PF: *Pseudomonas fluorescens.*

In order to explore the spectral variation in an unsupervised manner, a PCA model was developed from the mean spectra obtained from the masked image of each sample in the calibration set. Figure 3 shows the loadings and score plots of the first two PCs representing over 85% variance in the FTIR data and more than 98% variance of the VNIR and SWIR data. Spectra of the validation and test set were projected along the PC loading to produce the score values, as depicted by diamond-shaped markers. It is apparent from the PC score plots that the FTIR and VNIR mean spectra allow for a somewhat better separation between GP and GN types, compared with SWIR. GN samples tend to have positive score values on PC2, while GP samples mostly locate at the negative axis of PC2. This could be explained by the fact that PC2 loading bears some resemblance to the spectral profile of the GN samples (see Fig. 2). It can also be observed that it is challenging to separate bacterial species of the same Gram type, consistent with the mean spectral profiles in Fig. 2. The separation of mean FTIR spectra according to species, as seen in the PC score plots in Fig. 3, is less impressive than has been reported in earlier studies, for example^8^ found good separation among PC scores of IR spectra measured for gram negative species when bacterial suspensions were dried onto gold coated glass slides; however, only 12 spectra were measured per drop. Our results indicate the high intra-species variability in the measured spectra of the independent biological replicates, which justifies the use of further chemometric analysis to investigate the capability of each reflectance technique for gram and sub-gram discrimination.

### Classification according to Gram type

Initial models for classification were built on mean spectra from each drop of bacterial suspension. The accuracy for each spectral imaging modality/chemometric method/wavelength range when applied to the validation and test sets is shown in Table 2. Clearly, the classification models built on microscopic FTIR spectra were superior to those built on macroscopic VNIR or SWIR data, achieving classification accuracies of 100% on the validation and test sets in some spectral domains. Despite the considerable measurement challenges involved in the present study (i.e. the use of bacterial suspensions dried onto stainless steel substrates, the high intra-species spectral variability and the larger number of independent biological replicates), the high accuracy of gram level classification reported is consistent with the results reported by^8^, in which mean FTIR spectra of bacterial colonies transferred onto IR transparent substrates were perfectly classified according to gram type.

**Table 2:** Model performances for discrimination between Gram-positive and Gram-negative types based on mean spectra. For each modality/spectral region, the model yielding maximum accuracy in the validation set is bolded.

Modelling	Modality	Spectral region	Pre-treatment	nLV	Accuracy			Mean class accuracy		
					Validation	Test1	Test2	Validation	Test1	Test2
PLS-DA	FTIR	1350–675 cm^−1^	SNV + SG1	2	100.0	100.0	89.5	100.0	100.0	91.7
		1722–910 cm^−1^	SNV + SG1	8	100.0	100.0	100	100.0	100.0	100
		3500–2600 cm^−1^	SNV + SG1	3	100.0	100.0	57.9	100.0	100.0	66.7
		4000–675 cm^−1^	SNV + SG2	7	100.0	100.0	100	100.0	100.0	100
	Macroscopic	406–997 nm	SNV	6	90.0	95.0	25.0	89.6	94.8	27.1
		951–2496 nm	SNV	2	60.0	57.5	50.0	62.5	57.3	52.1
		406–2496 nm	SNV + SG1	5	**100.0**	85.0	60.0	100.0	82.3	52.1
SVM	FTIR	1350–675 cm^−1^	SNV		100.0	100.0	97.4	100.0	100.0	97.9
		1722–910 cm^−1^	SNV		100.0	100.0	92.1	100.0	100.0	89.3
		3500–2600 cm^−1^	SNV		100.0	100.0	100	100.0	100.0	100
		4000–675 cm^−1^	SNV		100.0	100.0	100	100.0	100.0	100
	Macroscopic	406–997 nm	SNV		**100.0**	95.0	45.0	83.3	93.8	43.8
		951–2496 nm	SNV		55.0	52.5.0	55.0	58.3	56.3	58.3
		406–2496 nm	SNV		80.0	90.0	40.0	83.3	87.5	37.5

As for the macroscopic spectra, there was an apparent advantage in fusing the VNIR and SWIR data when PLS-DA modelling was applied to the validation set, leading to a classification accuracy of 100% in the validation set; however this was reduced to 85% in Test1 which further decreased to 60% for Test2, while the SVM built on VNIR data after SNV pre-treatment yielded 100% accuracy in the validation set and 95% accuracy in Test1, yet reduced to 45% on Test 2. The lower classification accuracies obtained when applying the calibration models built from macroscopic VNIR or combined VNIR-SWIR data to the second test set indicate potential model overfitting (especially for the SVM model) and a lack of discrimination ability based on the mean spectra. These results can be compared to a recent study in which it was shown that VNIR spectral imaging could be used to identify and quantify the presence of lentiviral particles in dried fluid samples^15^ and to previous work on VNIR microscopic imaging for the classification of live bacterial cells^31^.

Subsequently, models were built for the classification of the sample according to Gram type, using pixel spectra. The accuracy for each spectral imaging modality/chemometric method/wavelength range was calculated through random cross validation on the calibration set (see an example in Fig. S5 for FTIR data in the region 1350–675 cm^−1^), and the optimal pretreatment/number of latent variable as determined on the calibration set was then applied to the validation and test sets as reported in Table 3. Overall, for the models built on FTIR pixel spectra, model performance appears to be worse than is the case for the models built on mean spectra as shown in Table 2. This is due in part to the larger number of pixel spectra as compared to mean spectra, and is also related to the improved signal to noise ratio inherent in averaging over all pixels, which benefits the models based on mean spectra. However, in terms of real-life application, where there could be multiple bacterial species present on a surface, pixel-wise prediction would be desirable, therefore it is essential to also inspect the performance of pixel-wise models. It is found that the overall performance for validation, Test1 and Test2 are comparable for all cases, which are different from using mean spectra where in some cases the Test2 performance is considerably inferior to validation and Test1. This implies the use of pixel spectra improves the model’s generalization ability in response to unseen data. In terms of the microscopic FTIR data, the best performing model for gram type was found to be in the full wavenumber region 4000–675 cm^−1^, for a SVM model based on SNV pre-treated spectra, leading to an accuracy of 99.7% in the validation set and an accuracy of > 96% in the test sets. PLS-DA models built on FTIR pixel spectra resulted in lower, yet still high classification accuracies, for example, a 7 latent variable PLS-DA model built on spectra in the 1722–910 cm^−1^ range with SNV followed by second derivative pre-treatment resulted in an accuracy of 94.2% in the validation set and > 98% in the test sets. At the pixel level, the classification of GP/GN bacteria was generally lower for fused VNIR/SWIR or VNIR spectral imaging data as compared to FTIR, with a pixel based PLS-DA models built on VNIR or fused VNIR-SWIR data producing classification accuracies > 84% in the validation and test sets. Confusion matrices for the optimal classification models for Gram type are shown in Table S5.

**Table 3:** Model performances for discrimination between Gram-positive and Gram-negative types based on pixel spectra. For each modality/spectral region, the model yielding maximum accuracy in the validation set is bolded.

Modelling	Modality	Spectral region	Pre-treatment	nLV	Accuracy			Mean class accuracy		
					Validation	Test1	Test2	Validation	Test1	Test2
PLS-DA	FTIR	1350–675 cm^−1^	SNV + SG2	6	93.9	98.2	98.0	93.4	98.3	98.0
		1722–910 cm^−1^	SNV + SG2	7	94.2	98.9	98.0	93.7	99.1	98.0
		3500–2600 cm^−1^	SNV + SG2	7	**96.5**	99.2	93.6	96.8	99.2	93.6
		4000–675 cm^−1^	SNV + SG2	8	91.9	96.7	94.2	91.7	96.4	94.2
	Macroscopic	24,631–10,030 cm^−1^ (406–997 nm)	None	1	79.6	77.6	85.2	75.2	73.8	85.6
		10,515–4006 cm^−1^ (951–2496 nm)	None	1	70.0	74.8	73.4	71.1	76.3	73.6
		24,631–4006 cm^−1^ (406–2496 nm)	None	1	**88.7**	83.2	84.0	87.0	80.7	84.4
SVM	FTIR	1350–675 cm^−1^	SNV		98.0	98.6	96.1	98.9	98.6	96.2
		1722–910 cm^−1^	SNV		99.3	99.0	94.8	99.3	99.0	94.8
		3500–2600 cm^−1^	SNV		98.9	99.5	90.7	98.9	99.5	90.7
		4000–675 cm^−1^	SNV		**99.7**	99.4	96.6	99.7	99.5	96.5
	Macroscopic	24,631–10,030 cm^−1^ (406–997 nm)	None		88.5	90.8	80.2	91.2	88.4	80.4
		10,515–4006 cm^−1^ (951–2496 nm)	None		81.4	74.8	67.2	77.4	70.2	67.6
		24,631–4006 cm^−1^ (406–2496 nm)	None		**91.2**	92.0	73.4	91.2	89.9	73.7

**Figure 4:**
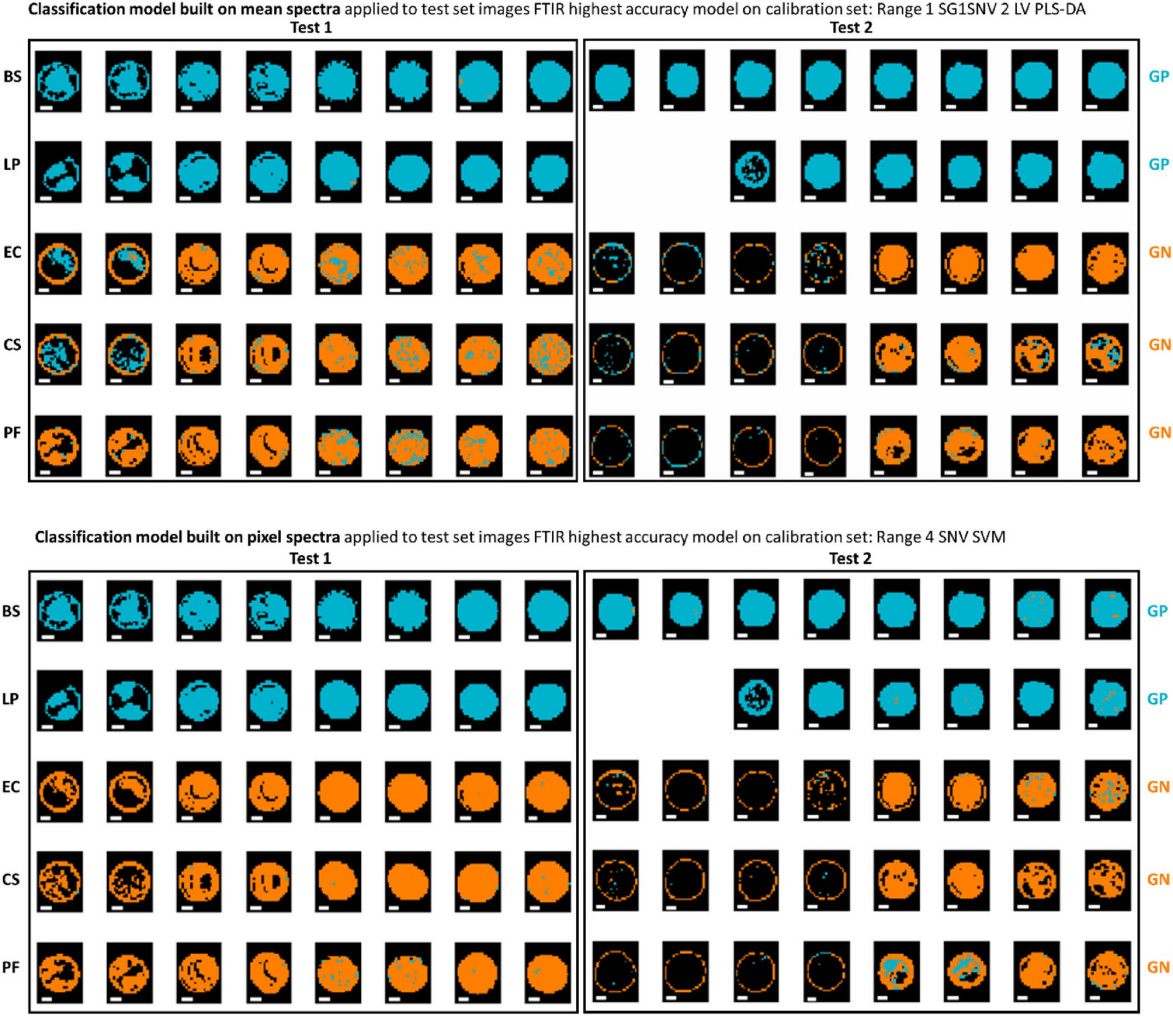
Classification maps for samples in the test sets obtained from the best mean (top panel) and pixel-level (bottom panel) FTIR models for GP/GN classification. Pixels classified as GP/GN are shown in blue or orange respectively. Each replicate has two drops. BS: *Bacillus subtilis*; LP: *Lactobacillus plantarum*; EC: *Escherichia coli*, CS: *Cronobacter sakazakii*; PF: *Pseudomonas fluorescens.* White scalebar indicates 1 mm (width) × 0.2 mm (height).

After model development, the best performing models, as selected by inspection of classification accuracy on the validation set, were applied to the pixels of all images in the test sets. Figure 4 shows the performance of the best prediction models built on both the mean and pixel FTIR spectra when applied to the pixels of the test set. In the prediction maps, pixels in blue colour are those predicted as being GP, while those in orange are the pixels predicted as being GN by the model. Although the models built on mean spectra appeared to perform well when applied to the mean spectra, they do not translate well to the pixel spectra, resulting in many misclassified pixels, particularly in the interior regions of the GN drops, as shown in Fig. 4. In contrast, the models built using the pixel spectra, which resulted in a very slightly lower accuracy in prediction i.e., 99 v’s 100%, perform much better at the pixel level, indicating the importance of building pixel-level models to predict at the pixel level (Tables 2 and 3). In general, all pixels of GP samples were correctly classified, while, even for models developed on pixel spectra, some pixels of the GN samples were incorrectly classified, and this misclassification mainly occurred sporadically in the central regions of the drops.

**Figure 5:**
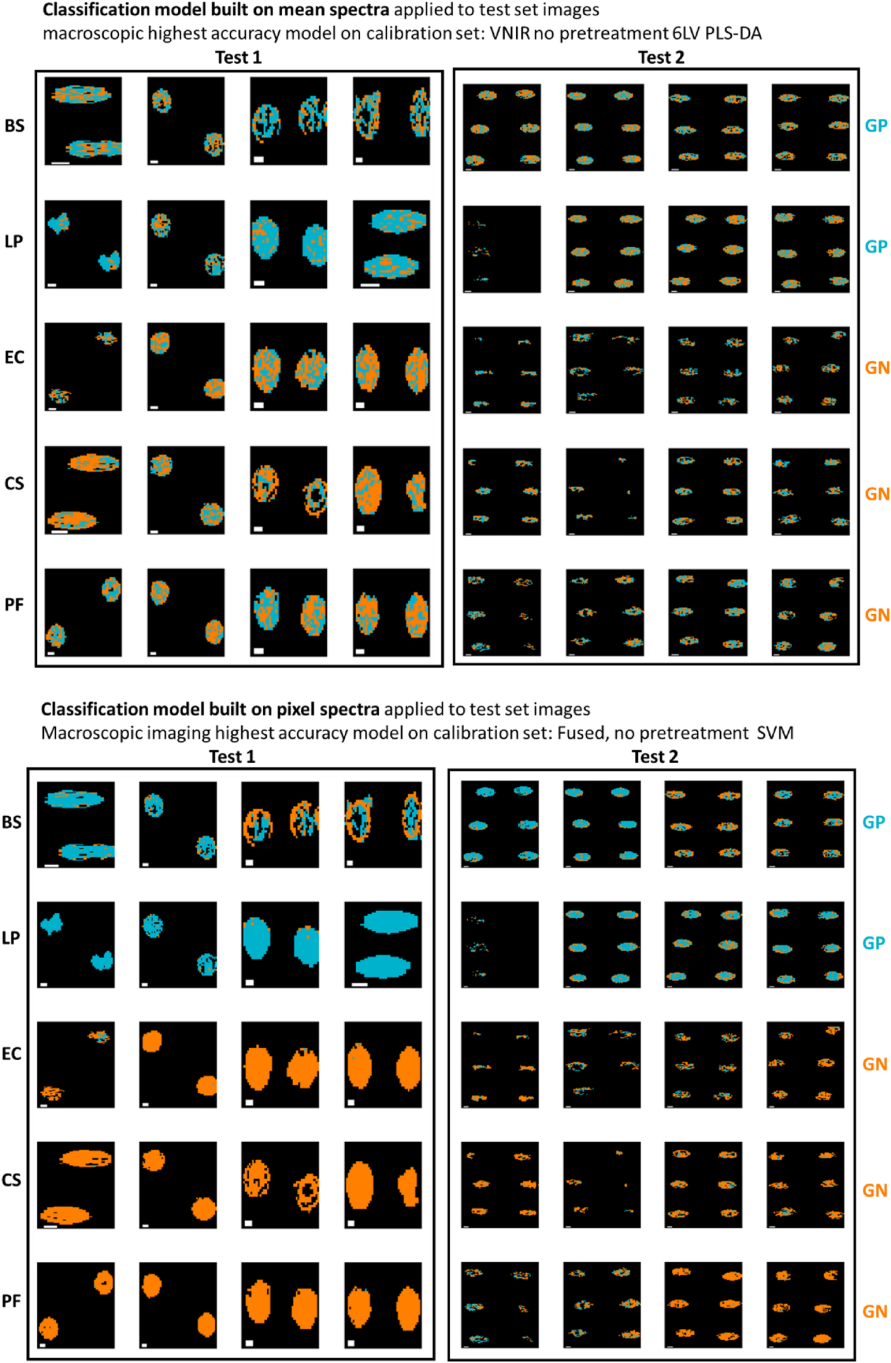
Classification maps for samples in the test set (reps 6, 9, 12, and 16) obtained from the best mean and pixel-level VNIR/SWIR models for GP/GN classification. Pixels classified as GP/GN are shown in blue or orange respectively. Each replicate has two drops. BS: *Bacillus subtilis*; LP: *Lactobacillus plantarum*; EC: *Escherichia coli*, CS: *Cronobacter sakazakii*; PF: *Pseudomonas fluorescens.* White scalebar indicates 1 mm (width) × 0.25 mm (height).

Similarly, prediction maps were generated from the macroscopic spectral imaging data, as shown in Fig. 5. Here, the advantage of using pixel-based classification is even more clear than was the case for the microscopic FTIR data: clearly a high level of pixel misclassification is found for the model built on mean spectra, while in general the classification is improved when using the model built on pixel spectra. In addition, in some cases, the masking process did not detect pixels in the central region (this was particularly evident for some of the GN Test 2 samples; this highlights variation in the drying process over experimental replicates.

### Classification within Gram type

#### Classification of Gram-positive samples

Having explored the capability for classification between GP and GN bacteria using the studied spectral imaging modalities, we then decided to investigate the considerably more challenging task of classification within gram types. Initially, the capability for classification between the two GP species (BS and LP) was studied. Models were built again using mean and pixel spectra, with the results as summarised in Tables 4 and 5. Here we can see that the task of classifying BS and LP was somewhat achievable using mean FTIR data, and PLS-DA models generally performed better than SVM models for this task. The best model performance on the validation set was found for a 6 LV PLS-DA model built on the 1350–675 cm^−1^ range after SNV followed by 1^st^ derivative Savitzky-Golay pre-treatment, resulting in an accuracy of 100% in both the validation and Test 1 sets. However, this model did not perform well on the second test set, with a massive reduction in classification accuracy to 31.3%. In contrast, a 9 LV PLS-DA model built on the 1722–910 cm^−1^ range after SNV followed by 2nd derivative Savitzky-Golay pre-treatment, resulted in a classification accuracy > 70% in the validation and both test sets. By comparison, a 6 LV model built on the raw VNIR data resulted in an accuracy of 100% in the validation set, and considerably lower accuracies (< 63%) in the test sets.

**Table 4:** Model performances for discrimination between gram-positive BS and LP based on mean spectra. For each modality/spectral region, the model yielding maximum accuracy in the validation set is bolded.

Modelling	Modality	Spectral region	Pre-treatment	nLV	Accuracy			Mean class accuracy		
					Validation	Test	Test2	Validation	Test	Test2
PLS-DA	FTIR	1350–675 cm^−1^	SNV + SG1	5	**100.0**	100.0	35.7	100.0	100.0	31.3
		1722–910 cm^−1^	SNV + SG2	9	100.0	87.5	71.4	100.0	87.5	75.0
		3500–2600 cm^−1^	MSC	10	100.0	87.5	57.1	100.0	87.5	50.0
		4000–675 cm^−1^	SNV + SG2	7	100.0	100	57.1	100.0	87.5	50
	Macroscopic	24,631–10,030 cm^−1^ (406–997 nm)	None	6	**100.0**	75.0	62.5	100.0	75.0	62.5
		10,515–4006 cm^−1^ (951–2496 nm)	None	1	62.5	68.8	37.5	62.5	68.8	37.5
		24,631–4006 cm^−1^ (406–2496 nm)	SG1	5	62.5	81.3	62.5	62.5	81.3	62.5
SVM	FTIR	1350–675 cm^−1^	SNV		**100.0**	87.5	42.9	87.5	87.5	37.5
		1722–910 cm^−1^	SNV		62.5	68.8	42.9	62.5	68.8	37.5
		3500–2600 cm^−1^	SNV		87.5	87.5	57.1	87.5	87.5	50.0
		4000–675 cm^−1^	SNV		25.0	56.3	42.9	25	56.3	37.5
	Macroscopic	24,631–10,030 cm^−1^ (406–997 nm)	SNV		62.5	81.3	50.0	87.5	81.3	50
		10,515–4006 cm^−1^ (951–2496 nm)	None		50.0	81.3	62.5	50	81.3	62.5
		24,631–4006 cm^−1^ (406–2496 nm)	SNV		**87.5**	75.0	37.5	87.5	75.0	37.5

**Table 5:** Model performances for discrimination between gram-positive BS and LP based on pixel spectra. For each modality/spectral region, the model yielding maximum accuracy in the validation set is bolded.

Modelling	Modality	Spectral region	Pre-treatment	nLV	Accuracy			Mean class accuracy		
					Validation	Test		Validation	Test	
PLS-DA	FTIR	1350–675 cm^−1^	SNV + SG2	9	**91.9**	93.2	39.0	91.9	93.5	34.0
		1722–910 cm^−1^	SNV + SG2	6	85.6	88.9	43.2	85.6	88.9	37.1
		3500–2600 cm^−1^	SNV + SG2	7	89.0	96.1	54.8	89.0	96.2	46.5
		4000–675 cm^−1^	SNV + SG2	7	75.5	92.5	41.4	75.5	92.4	35.9
	Macroscopic	24,631–10,030 cm^−1^ (406–997 nm)	None	7	59.0	65.2	47.6	57.4	65.3	46.7
		10,515–4006 cm^−1^ (951–2496 nm)	None	1	67.4	59.9	46.3	71.2	59.3	46.1
		24,631–4006 cm^−1^ (406–2496 nm)	None	10	**72.3**	64.9	49.4	73.0	65.0	48.5
SVM	FTIR	1350–675 cm^−1^	SNV		**93.0**	96.4	49.4	92.6	96.5	41.9
		1722–910 cm^−1^	SNV		88.7	92.3	55.5	88.7	92.7	47.0
		3500–2600 cm^−1^	SNV		92.6	95.3	59.8	92.6	95.5	50.6
		4000–675 cm^−1^	SNV		90.6	96.5	57.3	90.6	96.4	48.7
	Macroscopic	24,631–10,030 cm^−1^ (406–997 nm)	SNV		58.5	57.9	47.2	71.9	58.3	47.0
		10,515–4006 cm^−1^ (951–2496 nm)	None		64.9	62.5	52.9	67.4	62.2	52.7
		24,631–4006 cm^−1^ (406–2496 nm)	SNV		**70.2**	61.8	50.3	71.9	61.9	49.6

**Figure 6:**
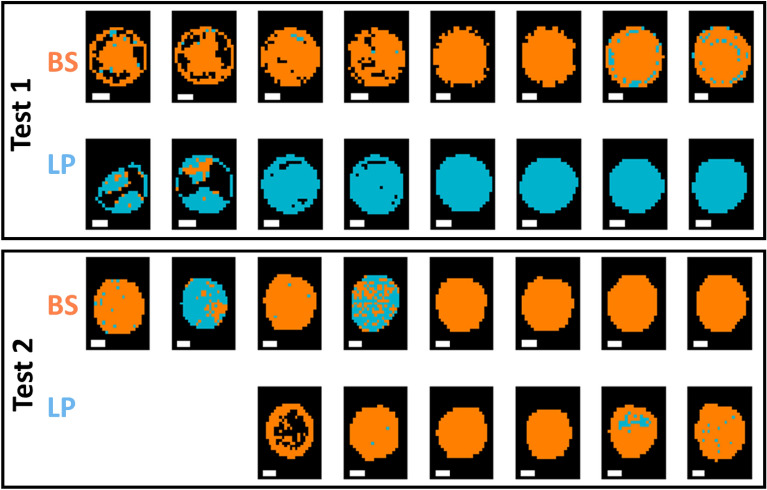
Classification maps for samples in the test set (reps 6, 9, 12, and 16) obtained from the best pixel-level FTIR models for GP species BS/LP classification. Pixels classified as BS/LP are shown in orange or blue respectively. Each replicate has two drops. BS: *Bacillus subtilis*; LP: *Lactobacillus plantarum.* White scalebar indicates 1 mm (width) × 0.2 mm (height).

**Figure 7:**
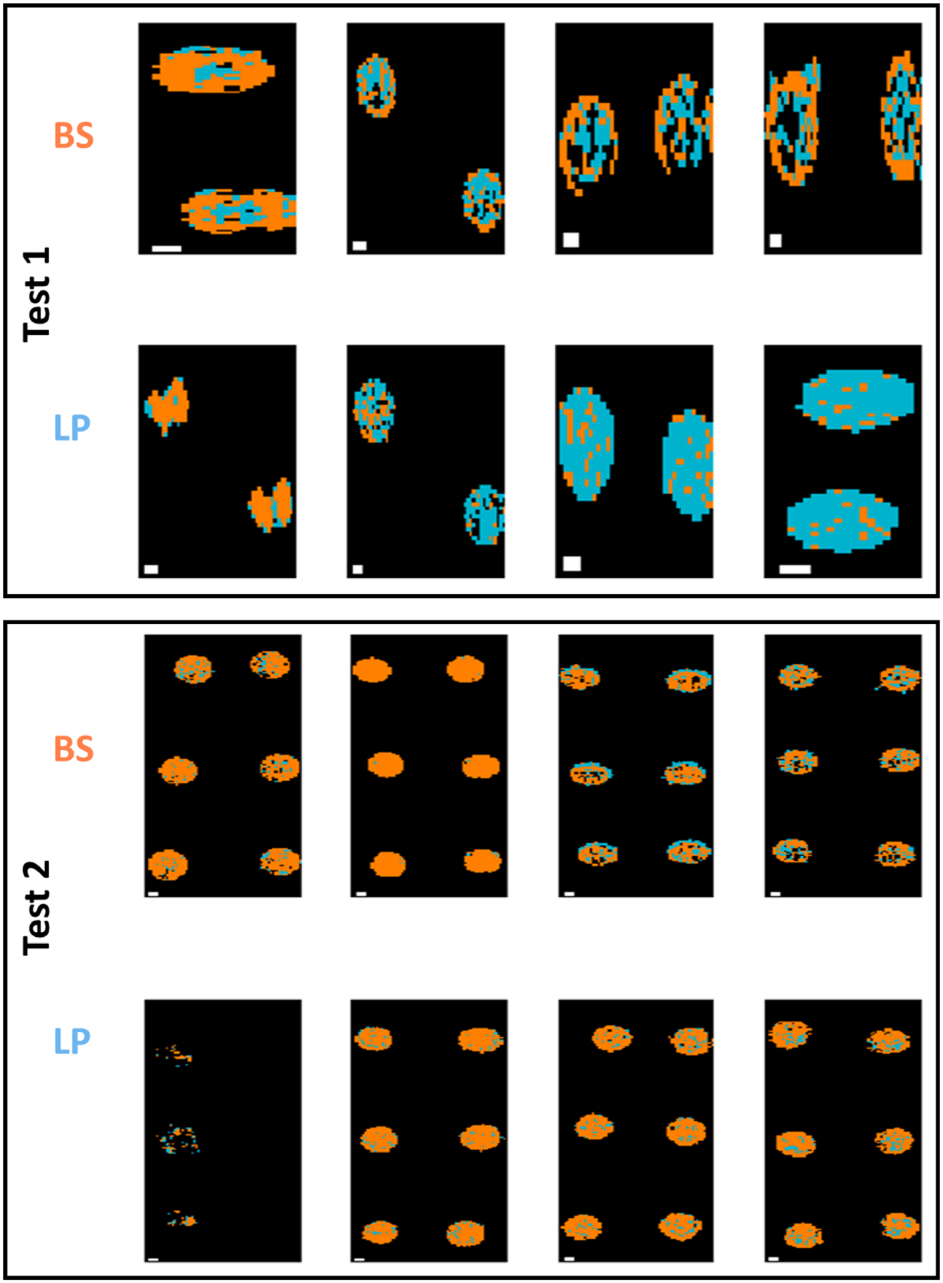
Classification maps for samples in the test set (reps 6, 9, 12, and 16) obtained from the best pixel level VNIR/SWIR models for GP BS/LP classification (pixels classified as BS/LP are shown in orange or blue respectively). Each replicate has two drops. BS: *Bacillus subtilis*; LP: *Lactobacillus plantarum*. White scalebar indicates 1 mm (width) × 0.25 mm (height).

As for classification models built on pixel spectra, as shown in Table 5, the FTIR microscopic data yielded a relatively high accuracy (93% for the validation set and 96% for Test 1) for an SVM model built on the 1350–675 cm^−1^ range after SNV pre-treatment, while the best performing model using the macroscopic data was based on Fused VNIR SWIR, where a 10 LV PLS-DA model built on pixel spectra without pre-treatment led to accuracies of 72% and 65% in the validation and test sets respectively. However, the performance of these models on the Test 2 dataset provided unacceptably low classification accuracies (50% for FTIR and 48% for the fused VNIR SWIR data). The corresponding prediction maps are shown in Figs. 6, 7 and Figs. S6, S7 for pixel and mean-level models, respectively. Clearly, the pixel level models provide prediction maps with fewer misclassified pixels, while the advantage of FTIR over fused VNIR SWIR is also clear, with fewer misclassified pixels in the FTIR prediction maps, and many misclassified pixels present in the fused VNIR SWIR prediction maps, including interior drop regions of some of the BS samples and the majority of pixels in 2 of the LP samples. Confusion matrices for the optimal classification models for Gram-positive samples are shown in Table S6.

#### Classification of Gram-negative samples

Following the investigation of the capability of each technique for sub-classification of the GP species examined, the GN samples were analysed to determine whether spectral imaging could be used to classify them, with the results shown in Tables 6 and 7. In general, all modalities and models performed relatively poorly for classification within the GN group. In terms of models built on the mean spectra, the best model within the FTIR data was an SVM model built on spectra in the 1722–910 cm^−1^ region with SNV pre-treatment, resulting in accuracies of 92%, 58% and 43% in the validation and test sets respectively. In contrast, a 9 LV PLS-DA model built on macroscopic VNIR data resulted in a modest 58% accuracy in the validation set and 54% accuracy in Test 1, reducing to 33% in Test 2. Surprisingly, the best SVM model on the macroscopic systems, which resulted in a poor 50% accuracy in the validation set, led to a reasonable accuracy of 71% in Test 1, but .reducing to 42% in Test 2. As for models built on pixel spectra, optimal performance on the validation set was found for an SVM built on FTIR spectra in the 3500–2600 cm^−1^ region, leading to accuracies of 62, 64% and 50% on the validation and test sets. Meanwhile, the optimal model within the macroscopic techniques was found for an SVM based on fused VNIR SWIR data after SNV pre-treatment, leading to low accuracies of 38.2, 42% and 35% on the validation and test sets respectively, although this was comparable to the SVM model built on the VNIR data alone (which led to accuracies of 38, 45% and 36% in the validation and test sets respectively).

**Table 6:** Model performances for discrimination between GN species EC, CS and PF based on mean spectra. For each modality/spectral region, the model yielding maximum accuracy in the validation set is bolded.

Modelling	Modality	Spectral region	Pre-treatment	nLV	Accuracy			Mean class accuracy		
					Validation	Test1	Test 2	Validation	Test1	Test2
PLS-DA	FTIR	1350–675 cm^−1^	SNV + SG2	12	66.7	66.7	50.0	66.7	66.7	50.0
		1722–910 cm^−1^	SNV	7	75	54.2	41.7	75	54.2	41.7
		3500–2600 cm^−1^	SNV + SG1	5	**75.0**	58.3	33.3	75.0	58.3	33.3
		4000–675 cm^−1^	SNV	7	66.7	45.8	37.5	66.7	45.8	37.5
	Macroscopic	24,631–10,030 cm^−1^ (406–997 nm)	SNV	9	58.3	54.2	33.3	58.3	54.2	33.3
		10,515–4006 cm^−1^ (951–2496 nm)	SG1	8	33.3	29.2	25	33.3	29.2	25
		24,631–4006 cm^−1^ (406–2496 nm)	SG2	8	33.3	41.7	41.7	33.3	41.7	41.7
SVM	FTIR	1350–675 cm^−1^	SNV		83.3	50.0	42.9	33.3	50.0	37.5
		1722–910 cm^−1^	SNV		**91.7**	58.3	42.9	91.7	58.3	37.5
		3500–2600 cm^−1^	SNV		33.3	45.8	57.1	33.3	45.8	50.0
		4000–675 cm^−1^	SNV		83.3	54.2	42.9	83.3	54.2	37.5
	Macroscopic	24,631–10,030 cm^−1^ (406–997 nm)	SNV		**50.0**	70.8	42.7	41.7	70.8	42.7
		10,515–4006 cm^−1^ (951–2496 nm)	SNV		33.3	25.0	33.3	33.3	25.0	33.3
		24,631–4006 cm^−1^ (406–2496 nm)	SNV		41.7	45.8	33.3	41.7	45.8	33.3

**Table 7:** Model performances for discrimination between GN species EC, CS and PF based on pixel spectra.

Modelling	Modality	Spectral region	Pre-treatment	nLV	Accuracy			Mean class accuracy		
					Validation	Test1	Test2	Validation	Test1	Test2
PLS-DA	FTIR	1350–675 cm^−1^	SNV + SG2	9	38.0	52.9	46.5	37.7	53.3	47.1
		1722–910 cm^−1^	SNV + SG1	7	53.3	52.4	38.1	53.1	52.7	38.6
		3500–2600 cm^−1^	SNV + SG2	5	**56.2**	55.4	31.5	56.7	55.8	31.9
		4000–675 cm^−1^	None	8	43.9	44.4	36.5	42.9	44.7	36.9
	Macroscopic	24,631–10,030 cm^−1^ (406–997 nm)	None	9	**41.7**	43.3	31.3	41.9	43.3	34.0
		10,515–4006 cm^−1^ (951–2496 nm)	None	13	34.3	28.1	37.4	31.4	28.2	31.9
		24,631–4006 cm^−1^ (406–2496 nm)	None	10	38.5	42.3	26.6	38.8	42.5	30.7
SVM	FTIR	1350–675 cm^−1^	SNV	0	57.4	63.3	49.4	61.0	63.5	41.9
		1722–910 cm^−1^	SNV	0	61.5	64.4	55.5	60.4	64.6	47.0
		3500–2600 cm^−1^	SNV	0	**62.0**	64.0	59.8	61.0	64.3	50.6
		4000–675 cm^−1^	SNV	0	54.4	66.0	53.2	53.2	66.1	48.7
	Macroscopic	24,631–10,030 cm^−1^ (406–997 nm)	SNV	0	38.0	45.2	35.9	38.3	44.8	36.4
		10,515–4006 cm^−1^ (951–2496 nm)	SNV	0	32.4	32.3	34.9	31.6	32.4	33.1
		24,631–4006 cm^−1^ (406–2496 nm)	SNV	0	**38.2**	42.1	34.4	38.3	42.0	35.5

For each modality/spectral region, the model yielding maximum accuracy in the validation set is bolded.

**Figure 8:**
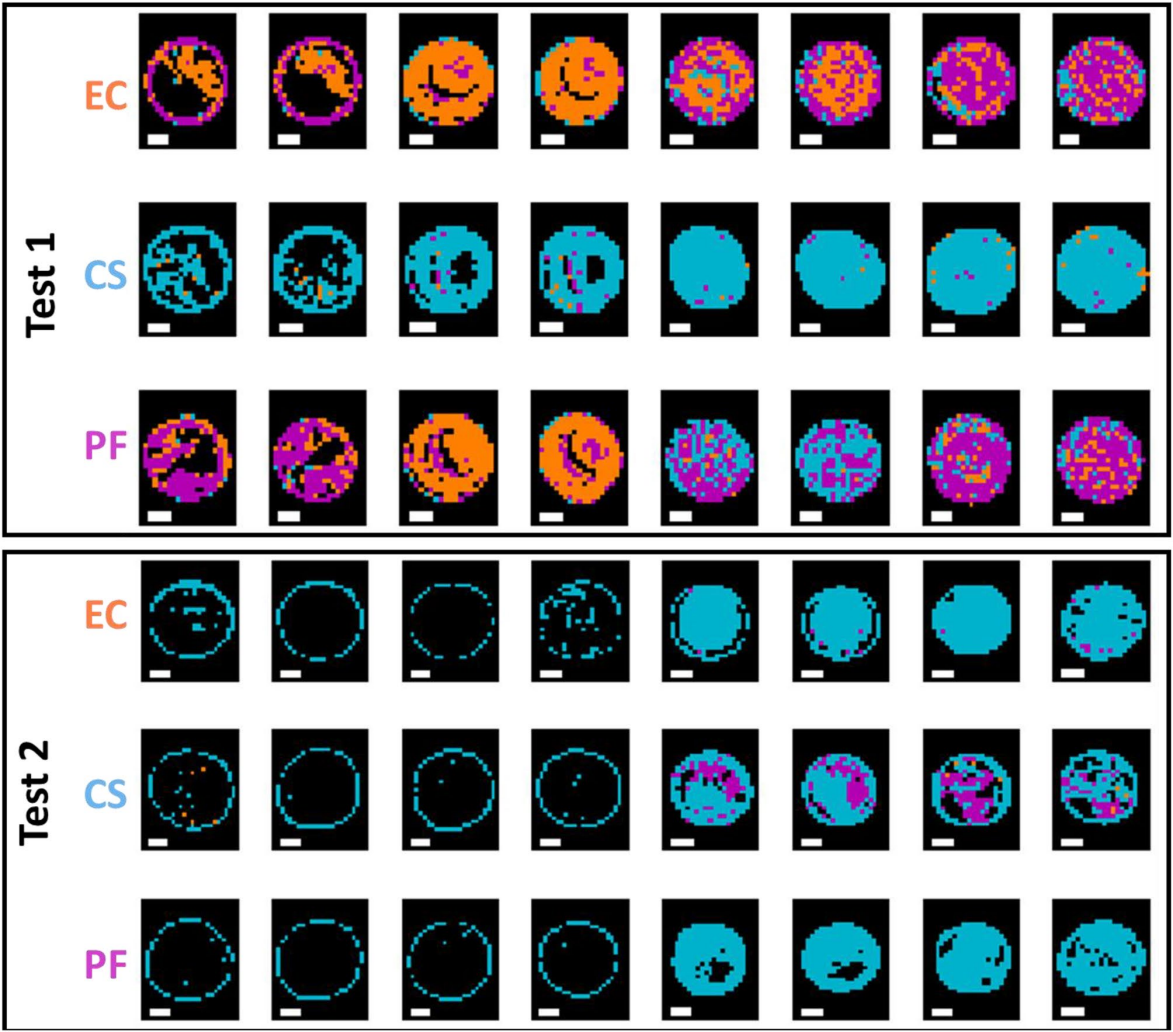
Classification maps for samples in the test set (reps 6, 9, 12, and 16) obtained from the best pixel-level FTIR models for GN EC/CS/PF classification (pixels classified as EC/CS/PF are shown in orange, blue or magenta respectively). Each replicate has two drops. EC: *Escherichia coli*, CS: *Cronobacter sakazakii*; PF: *Pseudomonas fluorescens.* White scalebar indicates 1 mm (width) × 0.2 mm (height).

**Figure 9:**
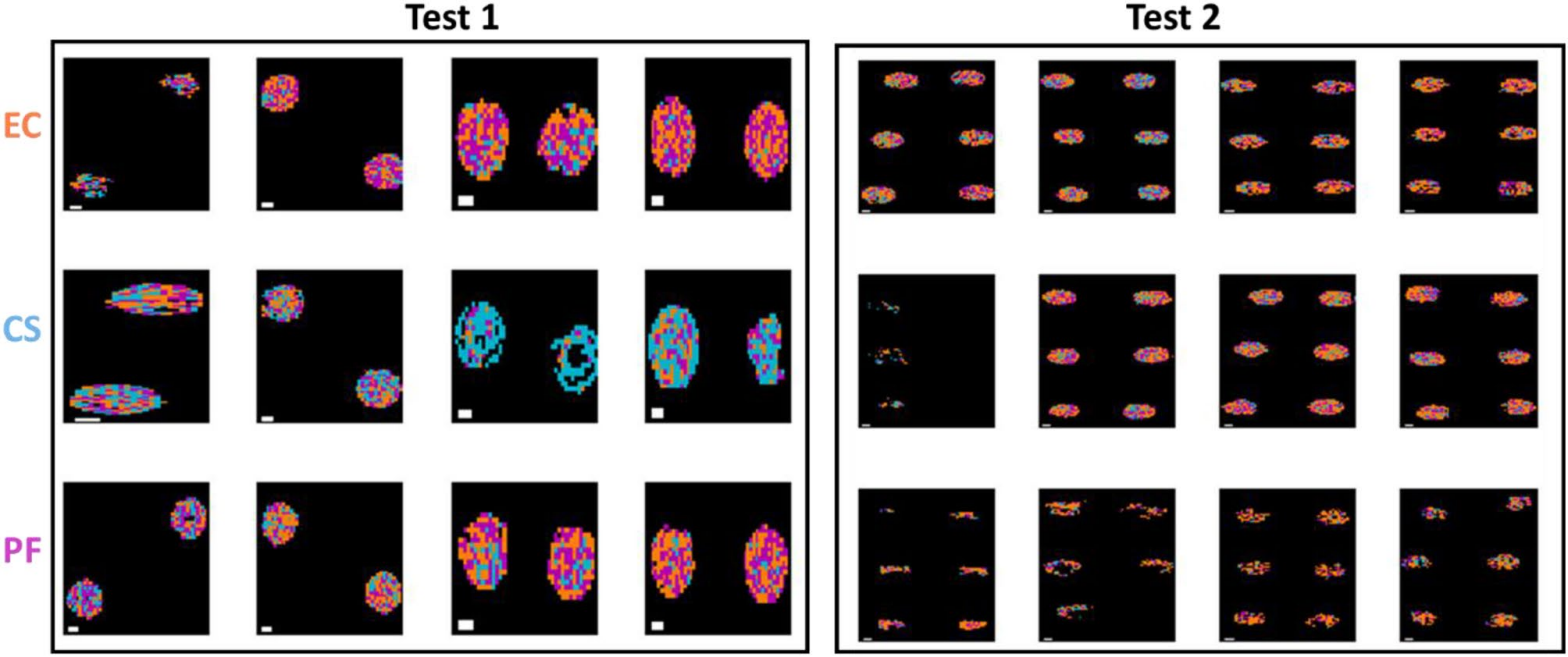
Classification maps for samples in the test set (reps 6, 9, 12, and 16) obtained from the best pixel level VNIR/SWIR models for GN species EC/CS/PF classification (pixels classified as EC/CS/PF are shown in orange, blue or magenta respectively). Each replicate has two drops. EC: *Escherichia coli*, CS: *Cronobacter sakazakii*; PF: *Pseudomonas fluorescens.* White scalebar indicates 1 mm (width) × 0.2 mm (height).

Despite the poor model performance for GN subclassification, the prediction maps corresponding to the optimal FTIR and VNIR/SWIR models, as shown in Figs. 8, 9 and Figs. S8, S9, are worth inspection. Here again, the advantage of pixel based classification models is clear, and it is interesting to examine the GN pixel classification maps based on FTIR data (Fig. 8), where it is clear that CS (middle row, blue pixels) is well classified for the validation and Test 1, but EC and PF cannot be clearly distinguished from each other. Meanwhile, the prediction maps for the models based on mean VNIR spectra (see Fig. S9) are very poor, and have the appearance of noise. Those based on pixel spectra (Fig. 9) are also poor; however, most of the CS samples appear to have more blue pixels than do the EC and PF, indicating a capability, albeit weak, of distinguishing these pixels from the EC and PF. Confusion matrices for the optimal classification models for Gram-negative samples are shown in Table S7.

### Deep learning

**Figure 10:**
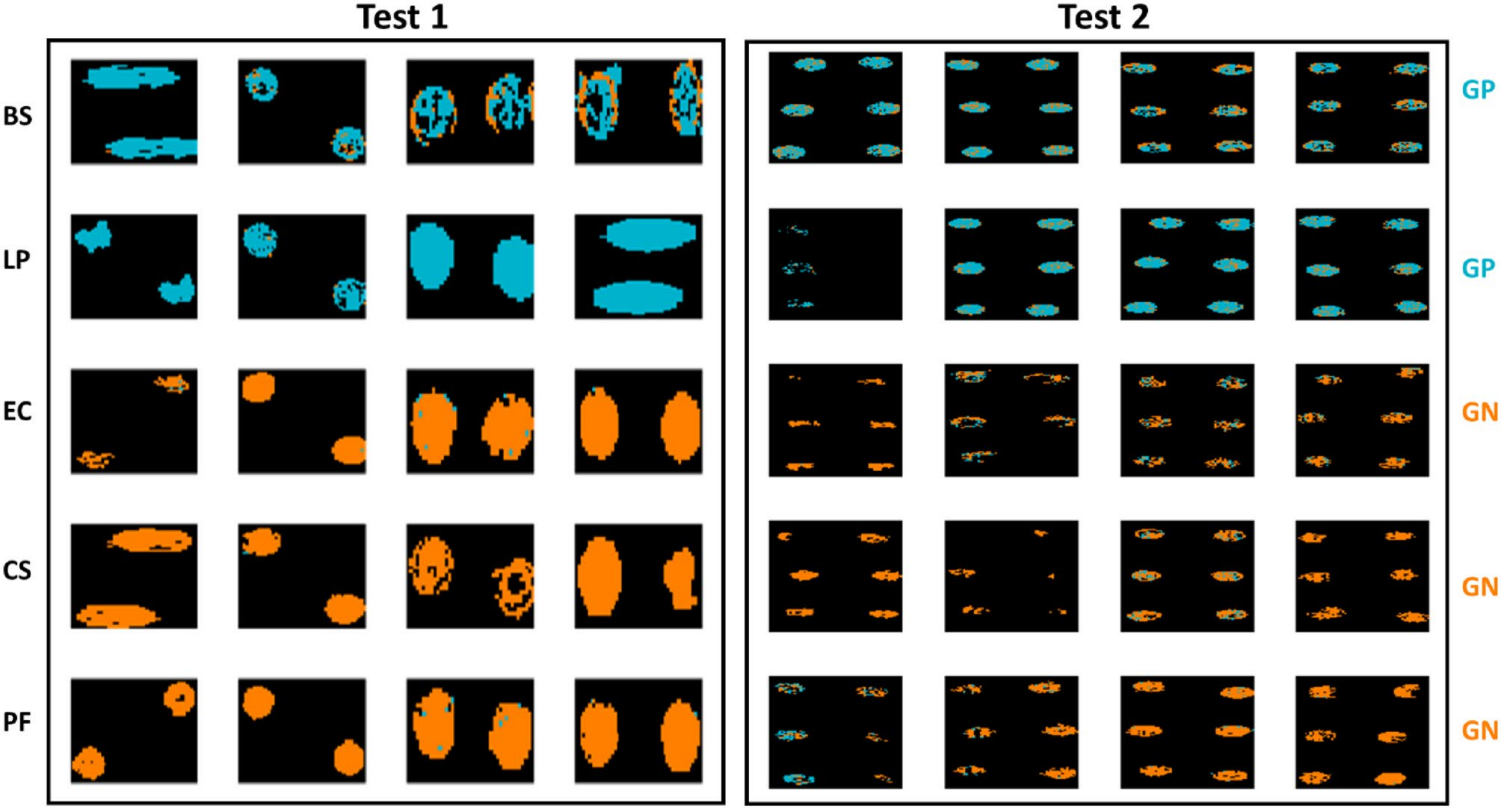
Classification maps for samples in the test set (reps 6, 9, 12, and 16) obtained from the best pixel-level PCA-LSTM VNIR/SWIR model for GP/GN classification. Pixels classified as GP/GN are shown in blue or orange respectively. Each replicate has two drops. BS: *Bacillus subtilis*; LP: *Lactobacillus plantarum*; EC: *Escherichia coli*, CS: *Cronobacter sakazakii*; PF: *Pseudomonas fluorescens.*

Due to the relatively poor performance of both PLS-DA and SVM models on the macroscopic spectral data, an advanced deep learning (DL) classification approach was applied to the data. DL models often lead to improved classification results due to their capability for extracting hidden and sophisticated structures/features within a data matrix while also supporting the extraction of both spectral and spatial features of spectral imaging data^21^. Herein, a hybrid approach of PCA and LSTM (PCA-LSTM) was also employed on the set of images obtained using the macroscopic VNIR and SWIR modalities. The classification accuracies for the developed PCA-LSTM models for the three classification problems studied in this work, i.e. classification between GP or GN, classification between the GP samples (BS/LP), and classification between the GN samples (EC/CS/PF), are summarised in Table 8. Clearly, the deep learning approach improved accuracy for the GP/GN classification task when applied to the validation and test set 1 data, reaching a classification accuracy of 97 and 96% on the validation and test sets (respectively) when applied to the fused VNIR/SWIR data, comparing very favourably to the SVM model developed on the same dataset (which resulted in accuracies of 91 and 92% on the validation and test sets); however, this model performed less well than the original PLS-DA model when applied to the second independent test set, reaching an accuracy of 80% (as compared with 84% for the PLS-DA model). Pixel level prediction maps for the PCA-LSTM GP/GN model are shown in Fig. 10. A subset of pixels distributed along the edge of drops of the GP BS samples are incorrectly classified as GN (see top right section of Fig. 10) whereas misclassified pixels in the GN class are less prominent.

**Table 8:** Model performances for PCA-LSTM models.

Classification	Spectral region	Accuracy			Mean class accuracy		
		Validation	Test1	Test2	Validation	Test1	Test2
**G+/G−**							
	406–997 nm	90.2	93.5	37.0	91.1	92.0	42.8
	951–2496 nm	80.2	83.3	72.6	83.5	83.9	72.5
	406–2496 nm	**97.6**	96.0	80.9	97.4	95.0	81.1
**BS/LP**							
	406–997 nm	65.9	69.2	62.3	66.3	69.3	63.1
	951–2496 nm	67.2	61.9	56.2	69.1	61.6	56.1
	406–2496 nm	**78.6**	70.7	65.7	81.8	70.4	65.5
**EC/CS/PF**							
	406–997 nm	28.2	29.2	27.9	27.7	29.3	27.5
	951–2496 nm	25.4	25.7	24.1	23.5	25.9	30.1
	406–2496 nm	**28.4**	28.6	23.1	27.9	28.8	28.4

For each spectral region, the model yielding maximum accuracy in the validation set is bolded.

**Figure 11:**
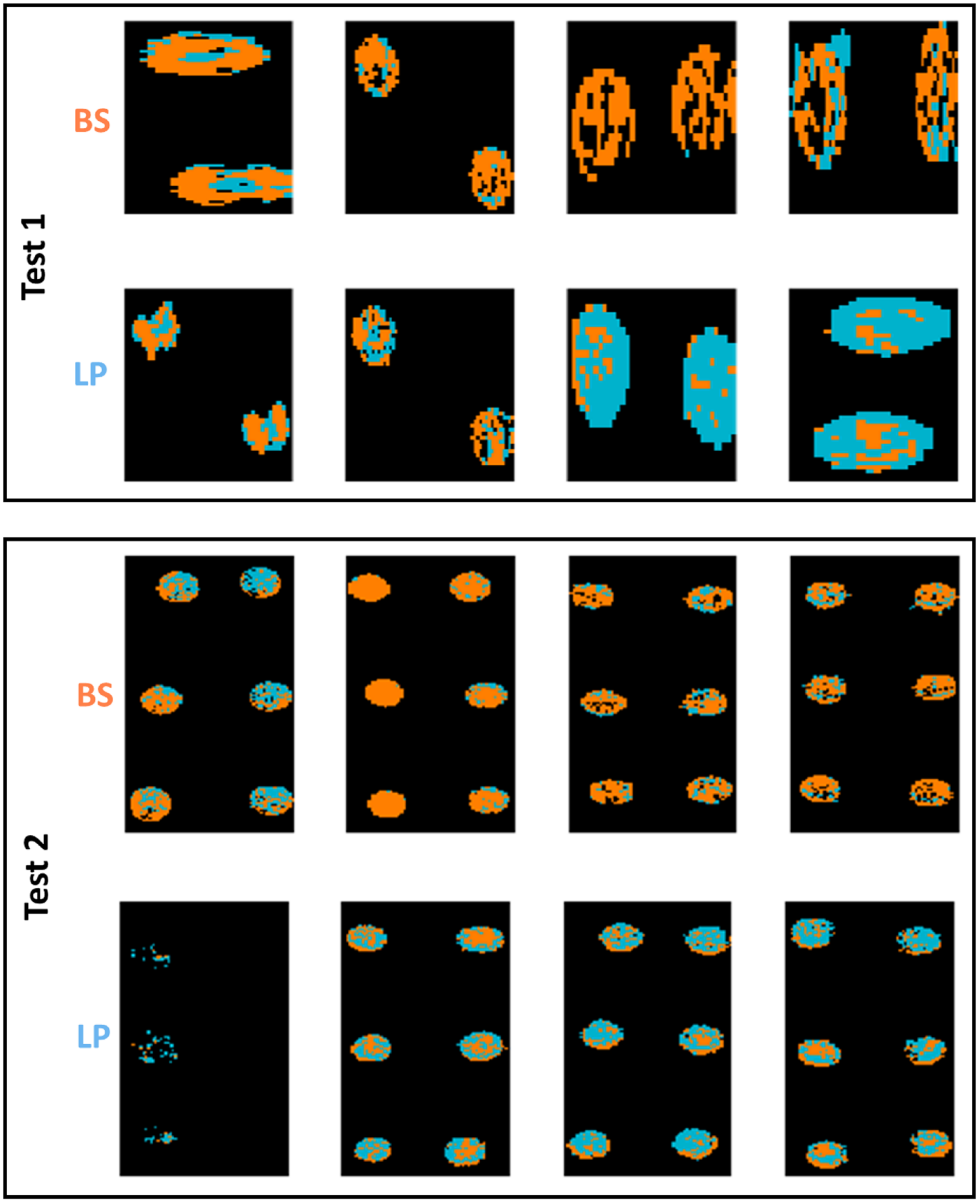
Classification maps samples in the test set (reps 6, 9, 12, and 16) obtained from the best pixel-level PCA-LSTM VNIR/SWIR model for GP BS/LP classification (pixels classified as BS/LP are shown in orange or blue respectively). Each replicate has two drops. BS: *Bacillus subtilis*; LP: *Lactobacillus plantarum*.

As for the more challenging task of discriminating between the GP species, the PCA-LSTM model built on fused data, with a classification accuracy of 71% when applied to Test 1, resulted in much higher classification accuracies than the PLS-DA model developed at the pixel level, which achieved an accuracy of 64%; however the PCA-LSTM model achieved similar classification accuracy of 65% when applied to Test 2. The corresponding pixel-level prediction maps are shown in Fig. 11. While most pixels are correctly predicted, and there is a significant improvement in the BS pixel-level prediction vis-à-vis the PLS-DA model prediction maps (Fig. 7), substantial incorrect classification can be seen in half of the LP test set samples (see Fig. 11 bottom left). Finally, the PCA-LSTM approach did not improve classification accuracy between the GN species, reaching a classification accuracy of 29% for fused data when applied to Test 1, as compared to the SVM model on the same dataset, which achieved an accuracy of 42%; when applied to Test 2, the PCA-LSTM model also performed worse than PLS-DA or SVM models, which seems to indicate overfitting of the deep learning approach due to the limited number of biological replicates.

## Conclusions

This work compares three spectral imaging modalities i.e. microscopic FTIR, macroscopic VNIR, and SWIR, in reflectance, for the discrimination of multiple bacterial species deposited and dried onto stainless steel. Classification models were developed at an object and pixel level. For the object level models built on mean spectra, two methods were compared for their classification accuracy, PLS-DA, and SVM. In addition to these two approaches, a deep learning method (PCA-LSTM) was applied to the pixel level models developed on macroscopic VNIR/SWIR data. In terms of object-level classification, PLS-DA and SVM were comparable in terms of model performance; however, SVM models generally outperformed PLS-DA when built on pixel spectra. PCA-LSTM models generally outperformed both SVM and PLS-DA; however,the PCA-LSTM requires hundreds of learnable parameters (see Fig. S2), the number of which is higher than the independent test samples but comparable to the number of pixels in each set and can thus lead to overfitting. Object-level models developed on mean spectra resulted in higher overall accuracies than those built on pixel spectra, however, they did not translate well to pixel-level classification, resulting in a higher proportion of misclassified pixels.

Overall, we found microscopic FTIR reflectance to be a more powerful technique than either of the macroscopic methods tested. Classification modelling performance validated and tested on independent replicates confirmed that the FTIR technique is capable of reliably discriminating between GP and GN bacteria, with accuracies in the independent test sets exceeding 98% for models developed on either mean or pixel spectra. However, macroscopic VNIR also achieved a reasonably high level of classification accuracy of 85% for PLS-DA models developed on pixel spectra and applied to independent test sets.

Further sub-classification of individual bacterial species within gram types indicated a lower robustness capability for classification between the GP *B. subtilis* and *L. plantarum* species with FTIR, with classification accuracies on the independent test set not exceeding 75%. The macroscopic techniques struggled with this classification task, reaching no higher than 62% accuracy for models developed on mean fused VNIR SWIR spectra. Bacteria from this work were grown in fresh TSB followed by washing and drying on stainless steel, the spectral signatures of which may be different from that of bacteria attached to equipment surfaces or in biofilms in the food industry. Therefore, future work is planned to validate and update the established models to facilitate the application in real-life. Overall, this work highlights the importance of validating with independent test sets, especially in the case of biological samples with high variability. Furthermore, more bacterial species that are relevant to foodborne illness (e.g. such as *Campylobacter*, *Salmonella* and *Listeria*) should be included in future work.

## Supplementary Information

Supplementary Information.

## Data Availability

The datasets generated and analysed during the current study are not publicly available due to potential commercial value but are available from the corresponding author on reasonable request.
