# Supplementary material for: Exploring the identification of multiple bacteria on stainless steel using multi-scale spectral imaging from microscopic to macroscopic
Source: Sci Rep. 2022 Sep 14;12:15412. doi: 10.1038/s41598-022-19617-3 (PMC9471055; doi:10.1038/s41598-022-19617-3)
Supplement: Supplementary file 1 — Supplementary Information. [file 41598_2022_19617_MOESM1_ESM.docx]

Table S1. Training options used in LSTM networks.

| GradientDecayFactor: 0.9000  SquaredGradientDecayFactor: 0.9990  Epsilon: 1.0000e-08  InitialLearnRate: 1.0000e-03  LearnRateSchedule: 'piecewise'  LearnRateDropFactor: 0.1000  LearnRateDropPeriod: 10  L2Regularization: 1.0000e-04  GradientThresholdMethod: 'l2norm'  GradientThreshold: 1  MaxEpochs: 100  MiniBatchSize: 128  Verbose: 1  VerboseFrequency: 50  ValidationFrequency: 100  ValidationPatience: 10  Shuffle: 'every-epoch'  CheckpointPath: ''  ExecutionEnvironment: 'auto'  WorkerLoad: []  OutputFcn: []  Plots: 'training-progress'  SequenceLength: 'longest'  SequencePaddingValue: 0  SequencePaddingDirection: 'right'  DispatchInBackground: 0  ResetInputNormalization: 1 |
| --- |

| Table S2**.** Cell counts of all sample replicates prepared on different dates. Missing data corresponds to samples for which microbial count was not carried out. | | | | | | |
| --- | --- | --- | --- | --- | --- | --- |
| **Replicates** | **Dates** (Day/Month/Year) | **Cell counts (logCFU/ml)** | | | | |
|  |  | **BS** | **LP** | **EC** | **CS** | **PF** |
| Reps 1, 2 | 11/11/2020 | 6.62 | 8.20 | 8.82 | 7.89 | 8.15 |
| Reps 3, 4 | 12/11/2020 | 6.34 | 7.88 | 8.66 | 7.14 | 8.27 |
| Reps 5, 6 | 18/11/2020 | 7.72 | 9.21 | 10.09 | 9.72 | 9.91 |
| Reps 7, 8 | 19/11/2020 | 8.08 | 9.66 | 10.75 | 10.58 | 9.73 |
| Reps 9, 10 | 17/12/2020 | - | - | - | - | - |
| Rep 11 | 05/02/2021 | 7.63 | 9.75 | 9.99 | 10.01 | 9.87 |
| Rep 12 | 12/02/2021 | 8.94 | 9.91 | 8.94 | 10.07 | 9.16 |
| Rep 13 | 17/02/2021 | 7.66 | 8.10 | 9.38 | 10.87 | 9.18 |
| Rep 14 | 18/02/2021 | 7.16 | 7.68 | 10.09 | 10.97 | 10.31 |
| Rep 15 | 19/02/2021 | 8.03 | 8.06 | 9.40 | 9.95 | 8.32 |
| Rep 16 | 26/02/2021 | 7.02 | - | 9.54 | 9.57 | 10.09 |
|  | 13/03/2021 | - | 8.43 | - | - | - |
| Rep 19 | 23/06/2022 | 8.88 | 8.21 | 10.06 | 10.72 | 10.12 |
| Rep 20 | 23/06/2022 | 8.58 | 9.85 | 10.08 | 9.58 | 9 |

Note: BS: *Bacillus subtilis*; LP: *Lactobacillus plantarum*; EC: *Escherichia coli*, CS: *Cronobacter sakazakii*; PF: *Pseudomonas fluorescens.*

Table S3. The number of pixels for each droplet from 15 replicates (Reps 1 & 3-16).

| Replicate |  | FTIR | | | | | VNIR/SWIR | | | | |
| --- | --- | --- | --- | --- | --- | --- | --- | --- | --- | --- | --- |
|  |  | BS | LP | EC | CE | PF | BS | LP | EC | CE | PF |
| Rep1 | Drop 1 | 161 | 90 | 82 | 113 | 66 | 64 | 56 | 47 | 80 | 234 |
|  | Drop 2 | 154 | 112 | 33 | 100 | 61 | 41 | 45 | 51 | 49 | 203 |
| Rep 3 | Drop 3 | 201 | 178 | 152 | 200 | 188 | 129 | 103 | 101 | 81 | 159 |
|  | Drop 4 | 196 | 167 | 170 | 210 | 188 | 128 | 118 | 124 | 112 | 152 |
| Rep 4 | Drop 5 | 148 | 117 | 148 | 185 | 198 | 195 | 18 | 104 | 101 | 147 |
|  | Drop 6 | 178 | 121 | 140 | 177 | 192 | 120 | 15 | 102 | 96 | 132 |
| Rep 5 | Drop 7 | 182 | 91 | 193 | 172 | 158 | 140 | 139 | 177 | 186 | 36 |
|  | Drop 8 | 180 | 106 | 180 | 185 | 144 | 144 | 61 | 167 | 128 | 43 |
| Rep 6 | Drop 9 | 179 | 117 | 167 | 169 | 184 | 212 | 105 | 89 | 187 | 139 |
|  | Drop 10 | 208 | 150 | 171 | 148 | 206 | 193 | 108 | 95 | 188 | 145 |
| Rep 7 | Drop 11 | 200 | 241 | 221 | 218 | 244 | 91 | 187 | 128 | 137 | 147 |
|  | Drop 12 | 183 | 243 | 231 | 228 | 229 | 54 | 197 | 132 | 112 | 145 |
| Rep 8 | Drop 13 | 210 | 159 | 246 | 213 | 265 | 104 | 85 | 110 | 110 | 144 |
|  | Drop 14 | 209 | 154 | 234 | 244 | 249 | 87 | 125 | 121 | 115 | 141 |
| Rep 9 | Drop 15 | 235 | 251 | 249 | 208 | 225 | 129 | 151 | 154 | 175 | 162 |
|  | Drop 16 | 207 | 262 | 239 | 232 | 275 | 131 | 132 | 153 | 205 | 163 |
| Rep 10 | Drop 17 | 202 | 210 | 239 | 266 | 212 | 94 | 179 | 166 | 163 | 174 |
|  | Drop 18 | 205 | 192 | 237 | 228 | 174 | 87 | 178 | 139 | 154 | 157 |
| Rep 11 | Drop 19 | 228 | 205 | 258 | 258 | 257 | 157 | 164 | 179 | 166 | 159 |
|  | Drop 20 | 241 | 236 | 270 | 320 | 275 | 142 | 164 | 162 | 167 | 134 |
| Rep 12 | Drop 21 | 263 | 264 | 279 | 276 | 261 | 111 | 170 | 176 | 136 | 148 |
|  | Drop 22 | 262 | 258 | 276 | 299 | 256 | 107 | 157 | 164 | 102 | 150 |
| Rep 13 | Drop 23 | 235 | 232 | 235 | 278 | 243 | 156 | 160 | 133 | 151 | 175 |
|  | Drop 24 | 257 | 195 | 233 | 273 | 252 | 152 | 149 | 118 | 129 | 157 |
| Rep 14 | Drop 25 | 222 | 246 | 197 | 180 | 256 | 65 | 174 | 154 | 175 | 165 |
|  | Drop 26 | 219 | 236 | 242 | 193 | 236 | 71 | 180 | 180 | 151 | 145 |
| Rep 15 | Drop 27 | 261 | 301 | 265 | 288 | 312 | 23 | 182 | 157 | 157 | 160 |
|  | Drop 28 | 281 | 301 | 294 | 278 | 298 | 111 | 190 | 165 | 152 | 158 |
| Rep 16 | Drop 29 | 301 | 215 | 249 | 303 | 304 | 198 | 168 | 197 | 218 | 176 |
|  | Drop 30 | 326 | 218 | 345 | 297 | 291 | 164 | 163 | 192 | 117 | 196 |
| Mean | - | 218 | 196 | 216 | 225 | 223 | 120 | 134 | 138 | 140 | 152 |

Table S4. The number of pixels of individual bacterial species in training, validation and test sets.

|  | FTIR | | | | VNIR/SWIR | | | |
| --- | --- | --- | --- | --- | --- | --- | --- | --- |
|  | Training | Validation | Test1 | Test2 | Training | Validation | Test1 | Test2 |
| BS | 3614 | 939 | 1981 | 2076 | 2290 | 391 | 1245 | 4143 |
| LP | 3186 | 947 | 1735 | 1410 | 2382 | 593 | 1154 | 3941 |
| EC | 3619 | 881 | 1975 | 1179 | 2465 | 547 | 1220 | 1997 |
| CS | 3831 | 976 | 1932 | 1129 | 2456 | 502 | 1328 | 2328 |
| PF | 3711 | 986 | 2002 | 1126 | 2696 | 629 | 1279 | 3247 |
| Total | 17961 | 4729 | 9625 | 6920 | 12289 | 2662 | 6226 | 15656 |

Table S5. The 10-fold cross-validation performance of SVM and PCA-LSTM for classification between Gram-positive and Gram-negative types based on pixel spectra.

| Modelling | Spectral region | Pre-treatment | Accuracy | Mean Class Accuracy |
| --- | --- | --- | --- | --- |
| SVM | 24631-10030 cm^-1^ (406–997 nm) | None | **90.8** | 89.4 |
|  | 10515-4006 cm^-1^ (951–2496 nm) | None | 76.3 | 73.2 |
|  | 24631-4006 cm^-1^ (406–2496 nm) | None | **93.1** | **92.3** |
| PCA-LSTM | 24631-10030 cm^-1^ (406–997 nm) | None | 91.5 | 91.5 |
|  | 10515-4006 cm^-1^ (951–2496 nm) | None | 84.0 | 83.2 |
|  | 24631-4006 cm^-1^ (406–2496 nm) | None | 93.8 | 93.8 |

| Table S5. Confusion matrices for the optimal classification models for Gram type. | | | | | | | | | | |
| --- | --- | --- | --- | --- | --- | --- | --- | --- | --- | --- |
| **Spectra** | **Modelling** | **Modality** | **Spectral region** | **Pre-treatment** | **nLV** | **Prediction map** | **Confusion matrices** | | |  |
|  |  |  |  |  |  |  |  | Actual Class | |  |
| Mean | PLS-DA | FTIR | 1350–675 cm^-1^ | SNV+SG1 | 2 | Fig 4 (top) |  | **GN** | **GP** |  |
|  |  |  |  |  |  |  | Predicted **GN** | 44 | 4 |  |
|  |  |  |  |  |  |  | Predicted **GP** | 0 | 30 |  |
| Pixel | SVM | FTIR | 4000–675 cm^-1^ | SNV |  | Fig 4 (bottom) |  | **GN** | **GP** |  |
|  |  |  |  |  | - |  | Predicted **GN** | 9089 | 254 |  |
|  |  |  |  |  |  |  | Predicted **GP** | 39 | 7163 |  |
| Mean | PLS-DA | Macroscopic | 406–2496 nm | SNV+SG1 | 5 | Fig 5 (top) |  | **GN** | **GP** |  |
|  |  |  |  |  |  |  | Predicted **GN** | 34 | 2 |  |
|  |  |  |  |  |  |  | Predicted **GP** | 12 | 12 |  |
| Pixel | SVM | Macroscopic | 406–2496 nm | None | - | Fig 5 (bottom) |  | **GN** | **GP** |  |
|  |  |  |  |  |  |  | Predicted **GN** | 10065 | 1334 |  |
|  |  |  |  |  |  |  | Predicted **GP** | 3334 | 7149 |  |

| Table S6. Confusion matrices for the optimal classification models for Gram-positive samples. | | | | | | | | | | |
| --- | --- | --- | --- | --- | --- | --- | --- | --- | --- | --- |
| **Spectra** | **Modelling** | **Modality** | **Spectral region** | **Pre-treatment** | **nLV** | **Prediction map** | **Confusion matrices** | | |  |
|  |  |  |  |  |  |  |  | Actual Class | |  |
| Pixel | SVM | FTIR | 1350–675 cm^-1^ | SNV |  | Fig 6 |  | **BS** | **LBP** |  |
|  |  |  |  |  | - |  | Predicted **BS** | 3578 | 479 |  |
|  |  |  |  |  |  |  | Predicted **LBP** | 1418 | 1727 |  |
| Pixel | PLS-DA | Macroscopic | 406–2496 nm | None | 10 | Fig 7 |  | **BS** | **LBP** |  |
|  |  |  |  |  |  |  | Predicted **BS** | 4239 | 1149 |  |
|  |  |  |  |  |  |  | Predicted **LBP** | 3786 | 1309 |  |

| Table S7. Confusion matrices for the optimal classification models for Gram-negative samples. | | | | | | | | | | |
| --- | --- | --- | --- | --- | --- | --- | --- | --- | --- | --- |
| **Spectra** | **Modelling** | **Modality** | **Spectral region** | **Pre-treatment** | **nLV** | **Prediction map** | **Confusion matrices** | | | |
|  |  |  |  |  |  |  |  | Actual Class | | |
| Pixel | SVM | FTIR | 3500–2600 cm^-1^ | SNV |  | Fig 8 |  | **EC** | **CS** | **PF** |
|  |  |  |  |  |  |  | Predicted **EC** | 961 | 1348 | 845 |
|  |  |  |  |  | - |  | Predicted **CS** | 56 | 2706 | 299 |
|  |  |  |  |  |  |  | Predicted **PF** | 623 | 1528 | 977 |
| Pixel | PLS-DA | Macroscopic | 406–997 nm | None | 9 | Fig 9 |  | **EC** | **CS** | **PF** |
|  |  |  |  |  |  |  | Predicted **EC** | 1799 | 434 | 984 |
|  |  |  |  |  |  |  | Predicted **CS** | 1815 | 997 | 844 |
|  |  |  |  |  |  |  | Predicted **PF** | 2581 | 712 | 1233 |


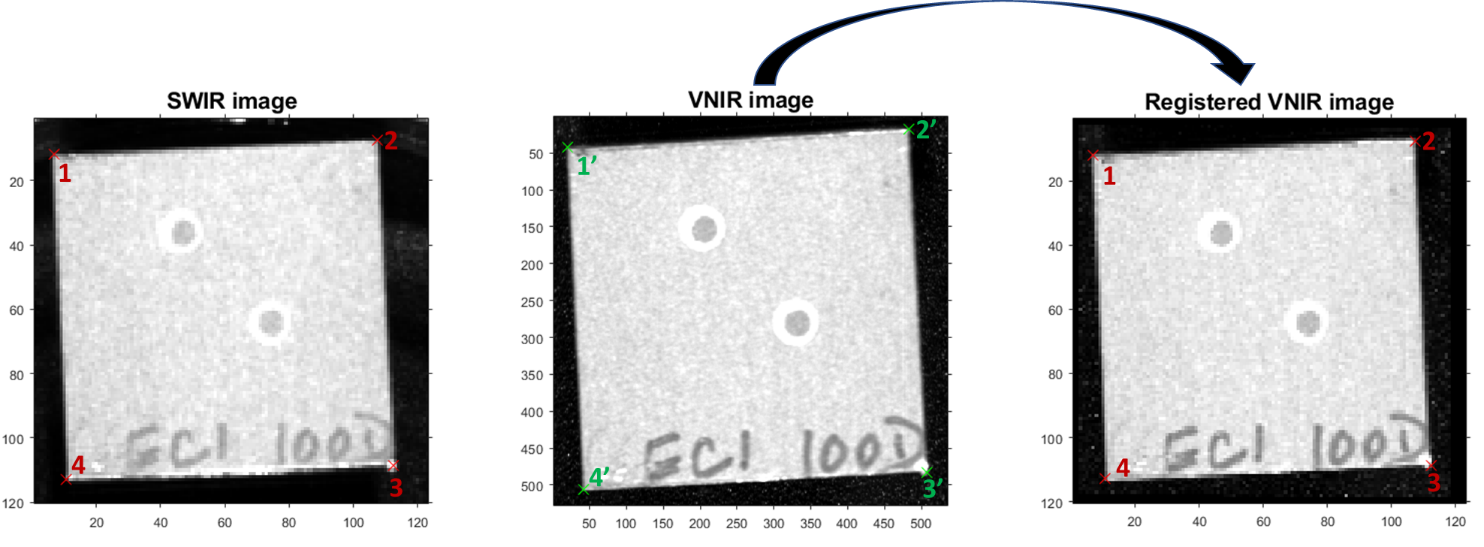


Figure S1. SWIR/VNIR image registration based on the four pairs of matching control points.


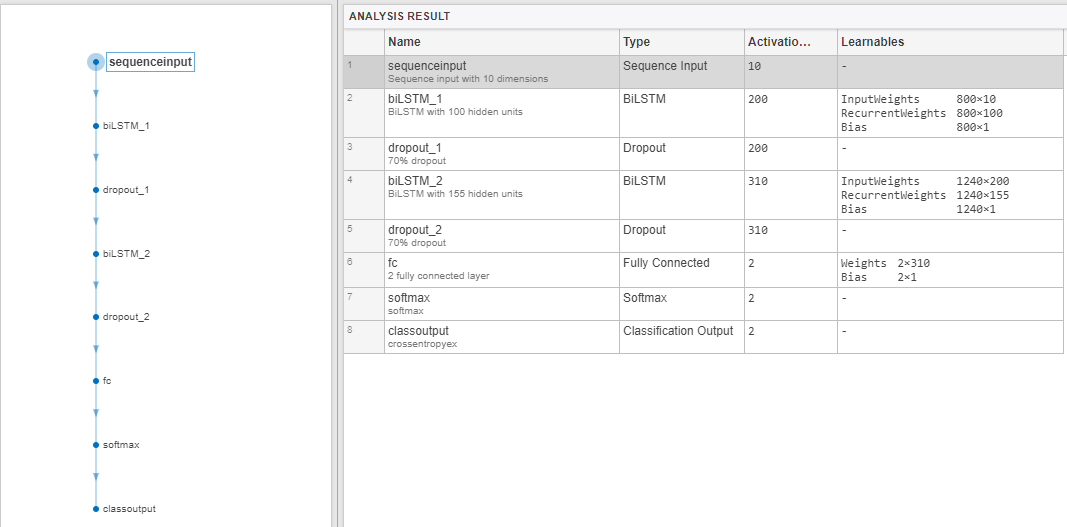


Figure S2. The structure and parameters for PCA-LSTM training for GP/GN classification.


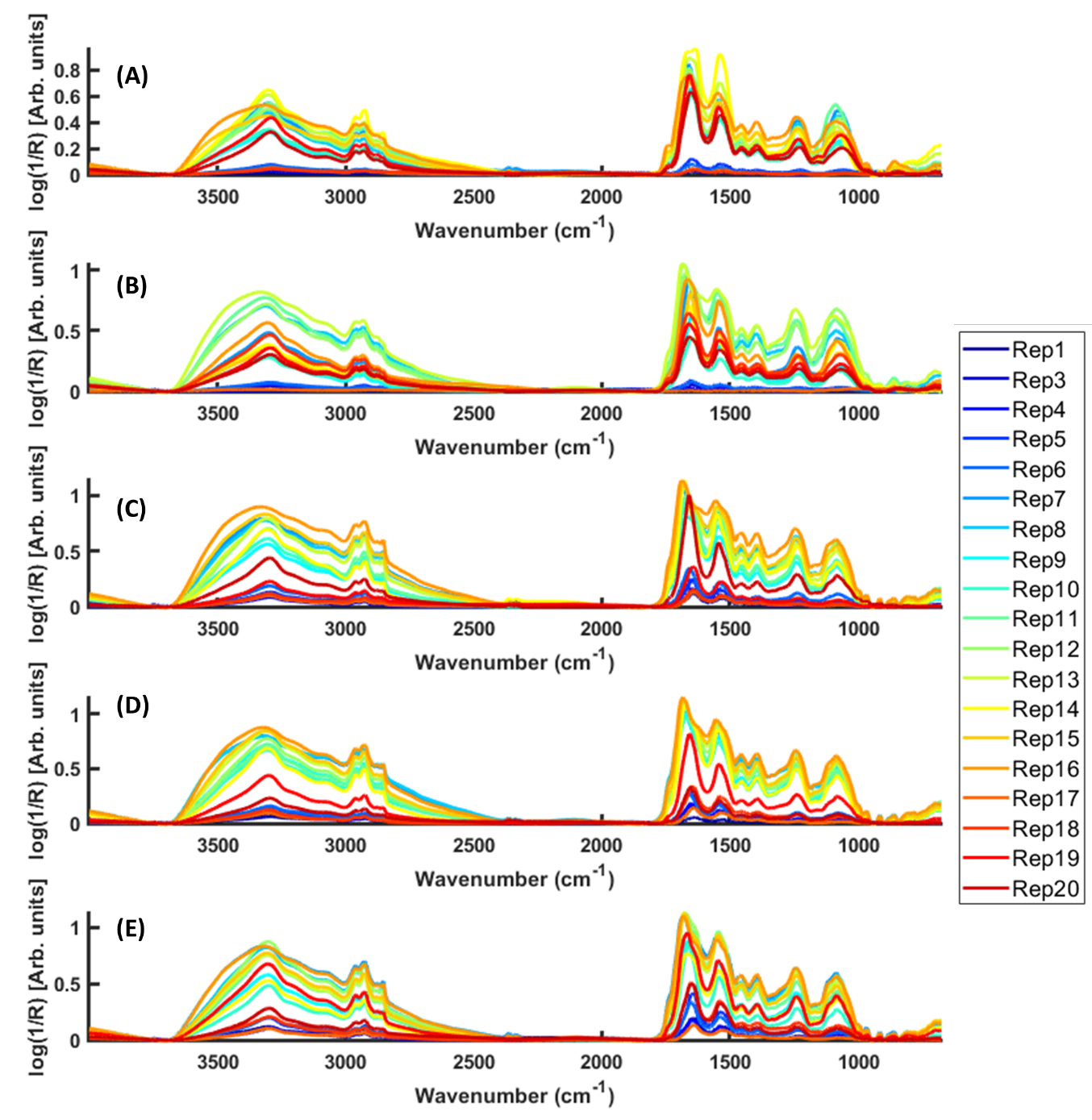


Figure S3. Mean FTIR spectra of each replicate (two drop images) of bacteria after applying asymmetric least squares smoothing to remove baseline). R = Reflectance. Arb.units = Arbitrary units.


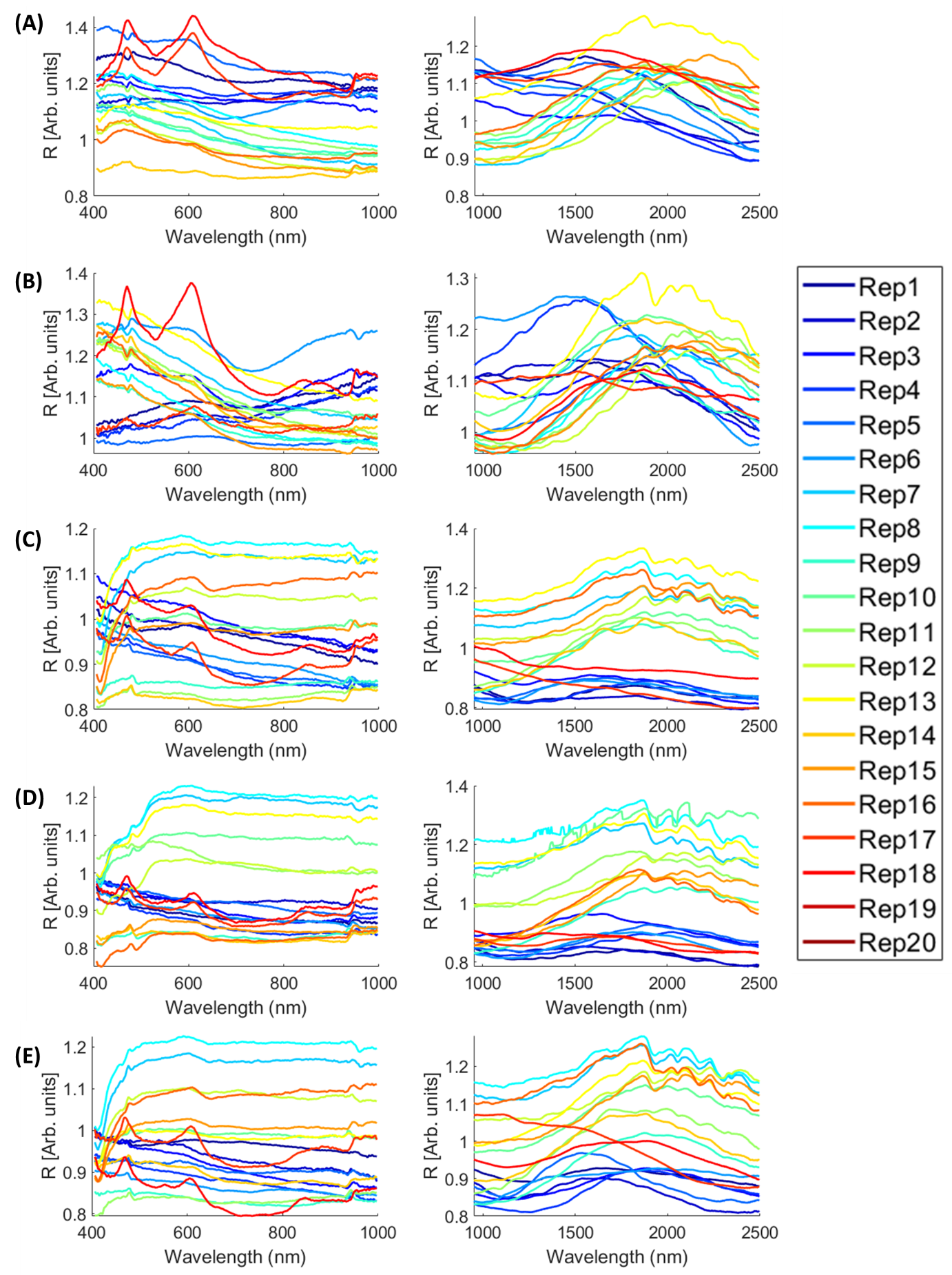
Figure S4. The mean spectra of each replicate of (A) *B. subtilis*, (B) *L. plantarum*, (C) *E. coli*, (D) *C. sakazakii,* and (E) *P. fluorescens*. R = Reflectance. Arb.units = Arbitrary units.

Figure S5. Cross validation results for GP/GN classification built on pixel spectra from calibration set of FTIR spectra (Range 1 = 1350–675 cm^-1^). Random cross validation was applied to the calibration set in which 70% of the spectra were randomly selected for model building and the remaining 30% were used for cross validation (the ‘randperm’ function in MATLAB was used to randomly permute the data). This process was repeated 100 times and the global and mean class accuracies, sensitivity, and specificity were calculated for each combination of spectral pre-treatment and number of latent variables. The optimal spectral pre-treatment and number of latent variables were selected based on consideration of the mean class accuracy, and product of sensitivity and specificity averaged over the 100 random folds on the calibration set. In this case, the optimal pretreatment was selected as 2^nd^ derivative & SNV, with 6 latent variables.


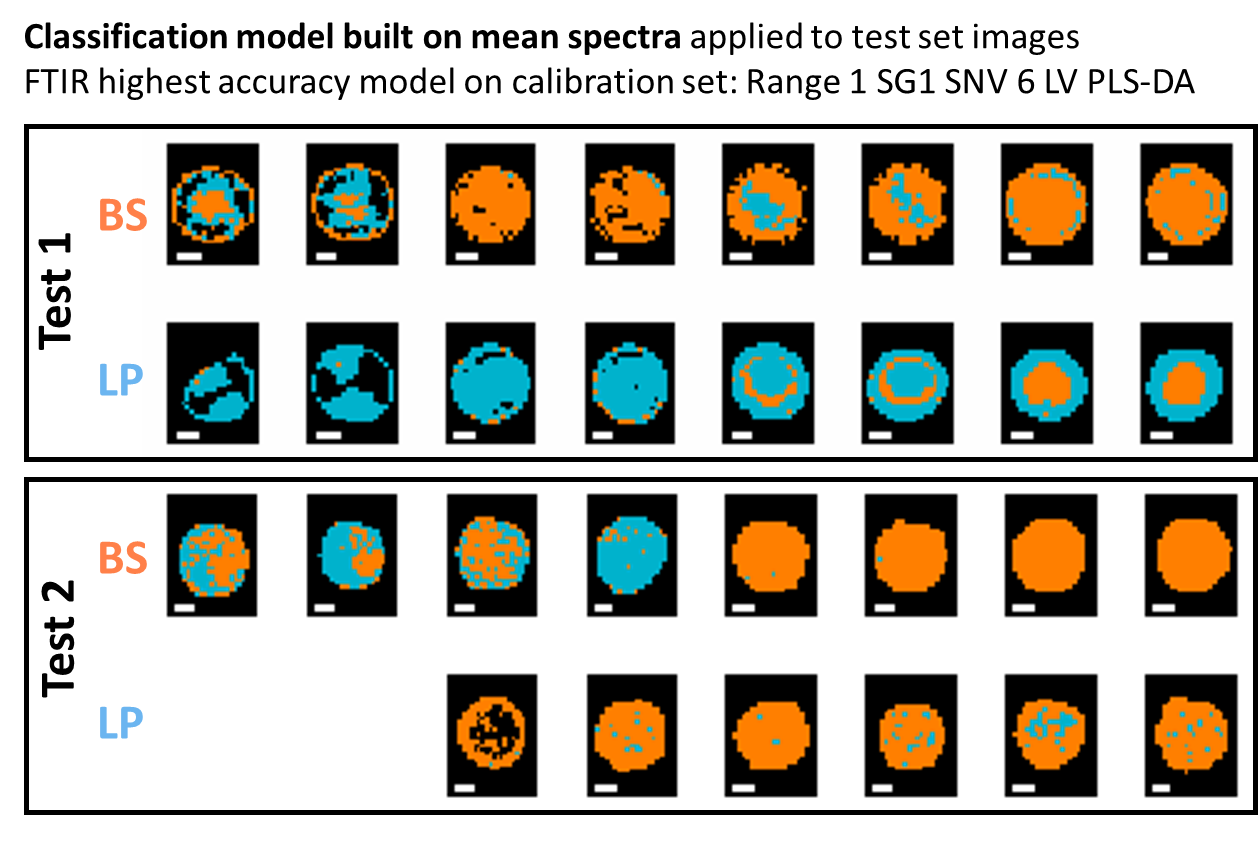


Figure S6. Classification maps for samples in the test set (reps 6, 9, 12, and 16) obtained from the best mean-level FTIR models for GP BS/LP classification. Each replicate has two drops. BS: *Bacillus subtilis*; LP: *Lactobacillus plantarum*.


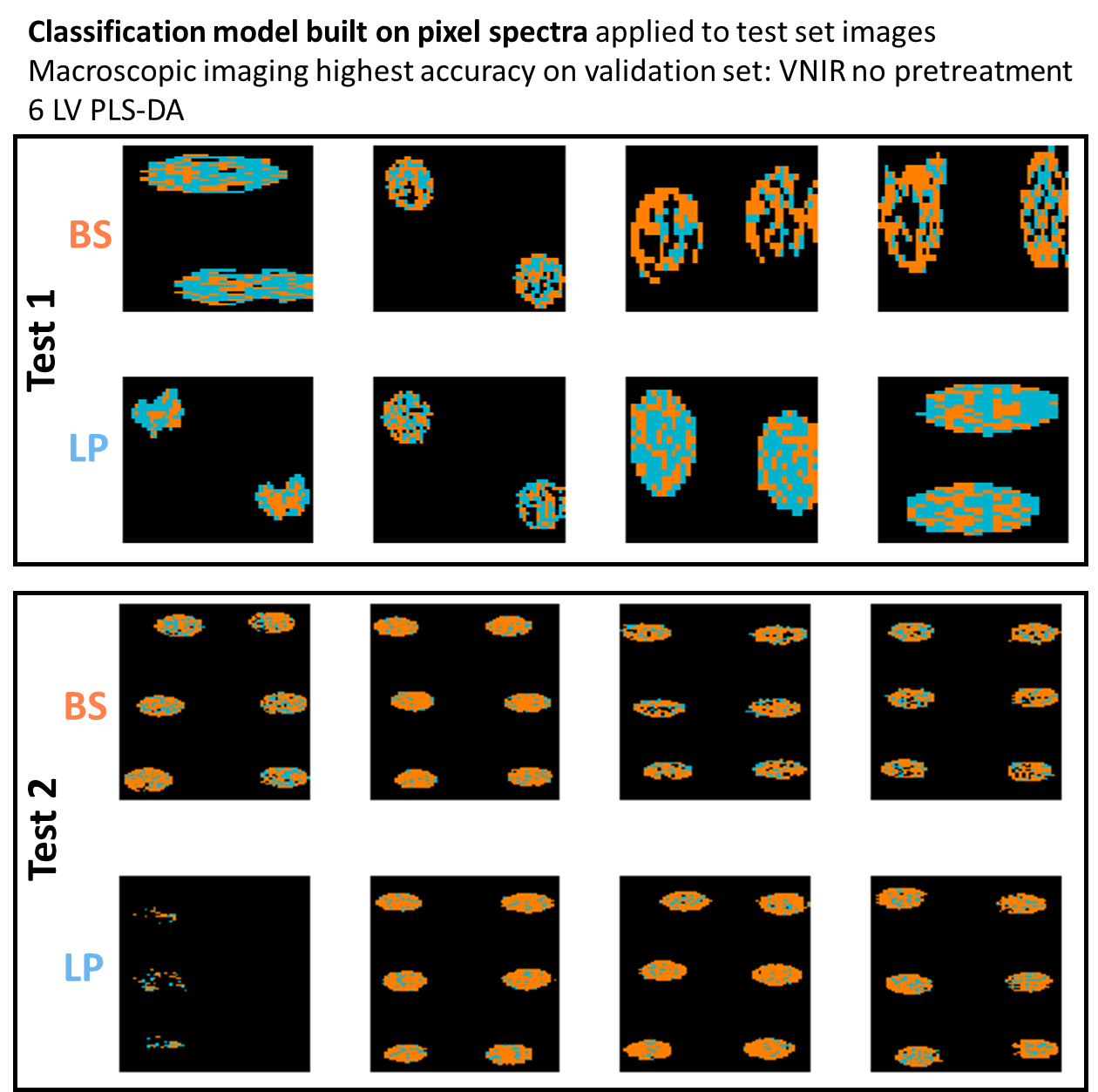


Figure S7. Classification maps for samples in the test set (reps 6, 9, 12, and 16) obtained from the best mean-level VNIR/SWIR models for GP BS/LP classification. Each replicate has two drops. BS: *Bacillus subtilis*; LP: *Lactobacillus plantarum*.


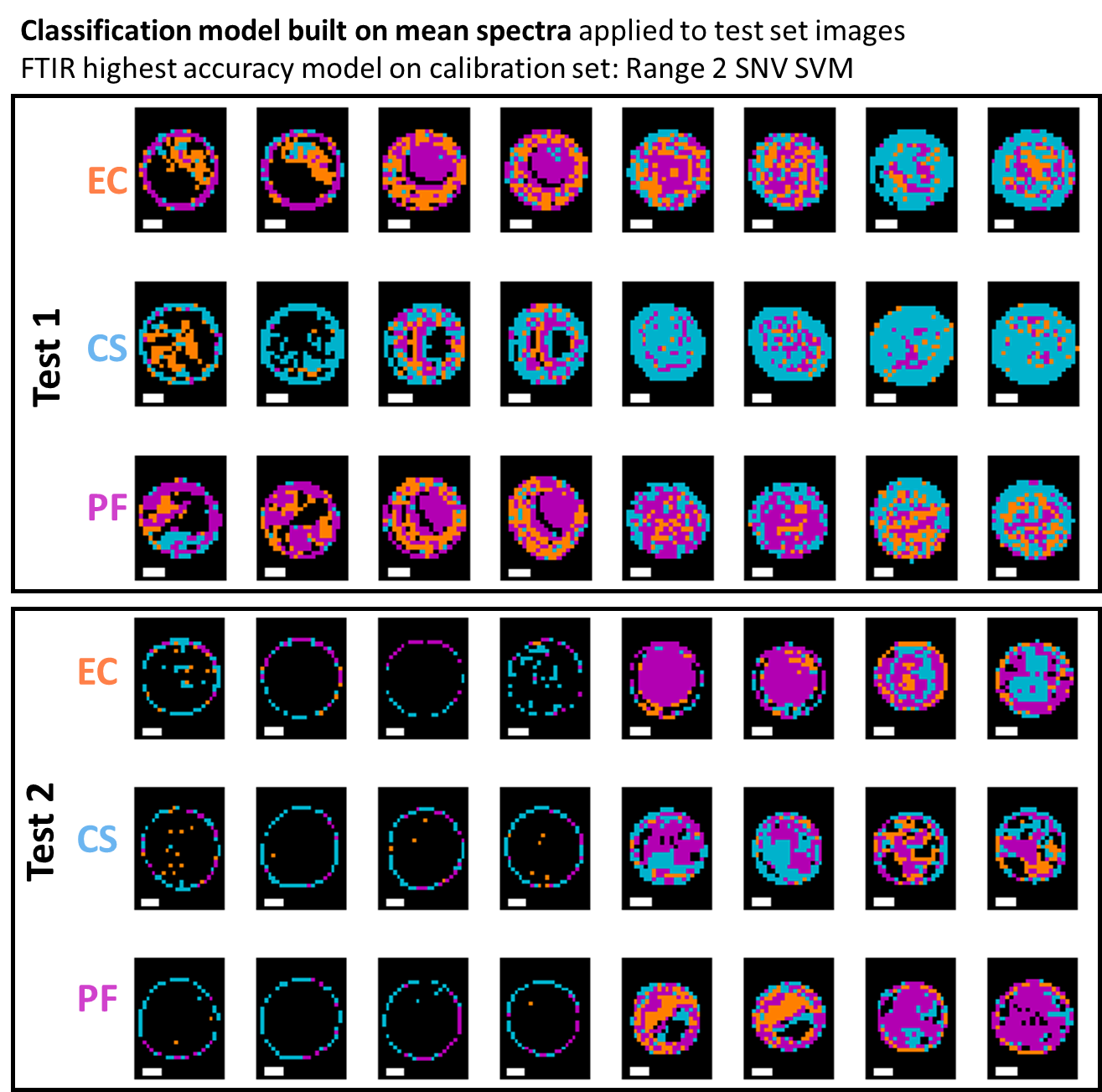


Figure S8. Classification maps for samples in the test set (reps 6, 9, 12, and 16) obtained from the best mean-level FTIR models for GN EC/CS/PF classification. Each replicate has two drops. EC: *Escherichia coli*, CS: *Cronobacter sakazakii*; PF: *Pseudomonas fluorescens.*


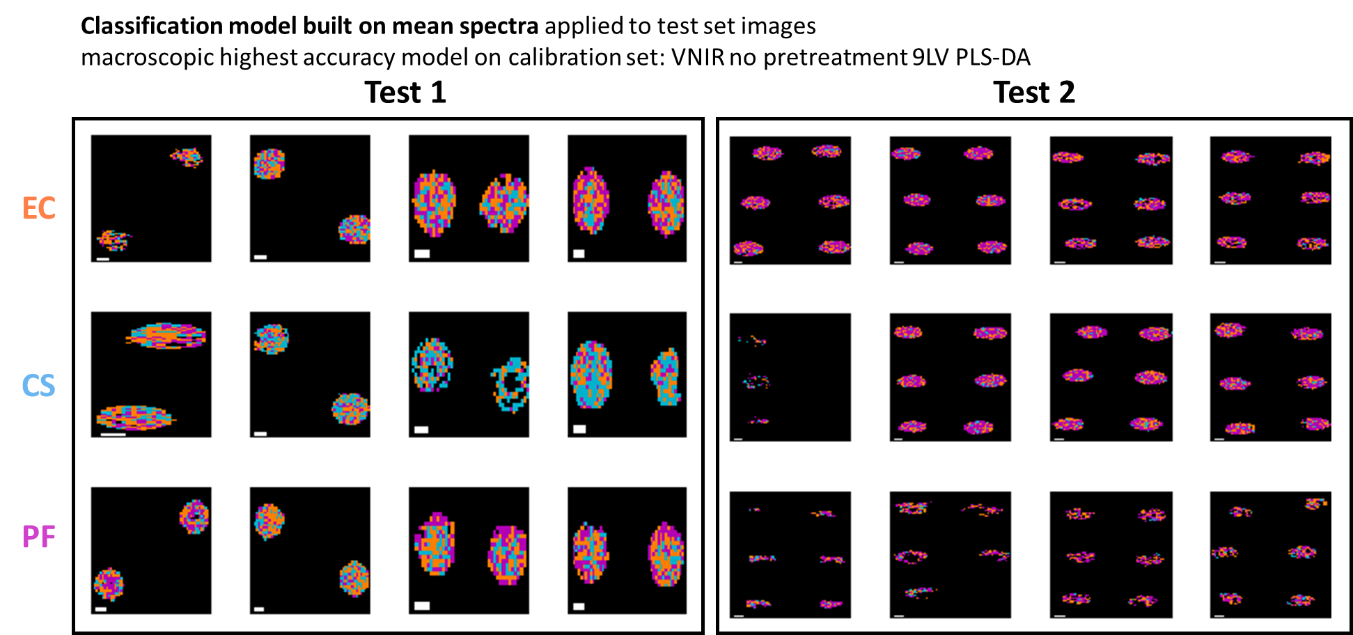


Figure S9. Classification maps for samples in the test set (reps 6, 9, 12, and 16) obtained from the best mean-level VNIR/SWIR model for GN EC/CS/PF classification. Each replicate has two drops. EC: *Escherichia coli*, CS: *Cronobacter sakazakii*; PF: *Pseudomonas fluorescens.*
